# Supplementary material for: Combined Omipalisib and MAPK Inhibition Suppress PDAC Growth
Source: Cancers (Basel). 2025 Mar 29;17(7):1152. doi: 10.3390/cancers17071152 (PMC11987824; doi:10.3390/cancers17071152)
Supplement: Supplementary file 1 [file cancers-17-01152-s001.zip › cancers-3471401-supplementary.pdf]

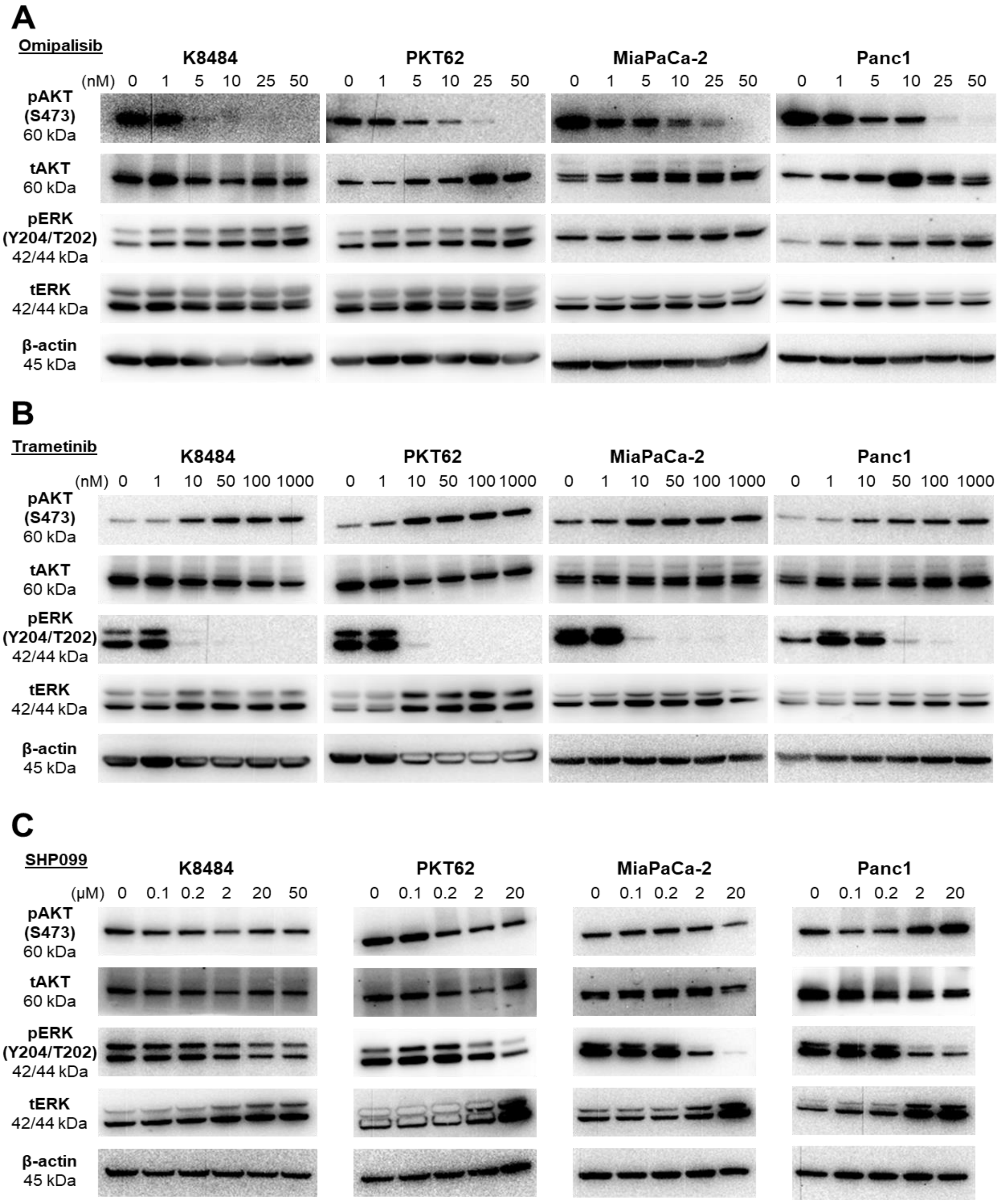

Supplemental Figure S1. Dose response of PDAC tumor cells to pathway inhibition via Omipalisib (A), Trametinib (B), or SHP099 (C). Western blots demonstrating inhibition and/or activation of either PI3K (pAKT compared to total AKT levels) or MAPK (pERK compared to tERK levels) pathways upon treatment for 24 hours (A, B) or 3 hours (C).

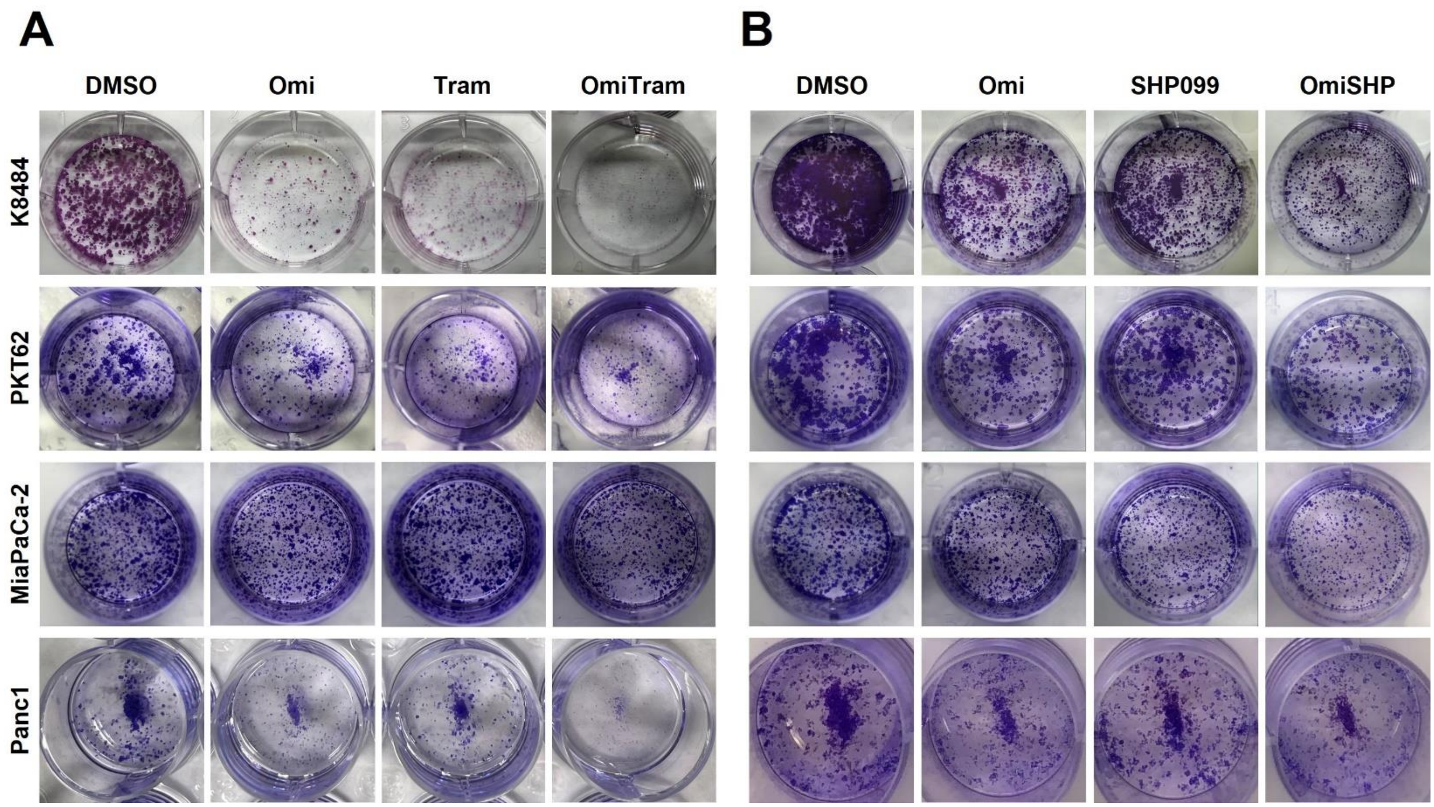

Supplemental Figure S2. Representative images of colony-forming assay at endpoint, as quantified in Figure 2. K8484 cells were treated with 5nM Omipalisib, 10 nM Trametinib, and/or 20 uM SHP099. PKT62 cells were treated with 5 nM Omipalisib, 10 nM Trametinib, and/or 2 uM SHP099. MiaPaCa-2 cells were treated with 5 nM Omipalisib, 5 nM Trametinib, and 20 uM SHP099. Panc1 cells were treated with 50 nM Omipalisib, 20 nM Trametinib, and 50 uM SHP099.

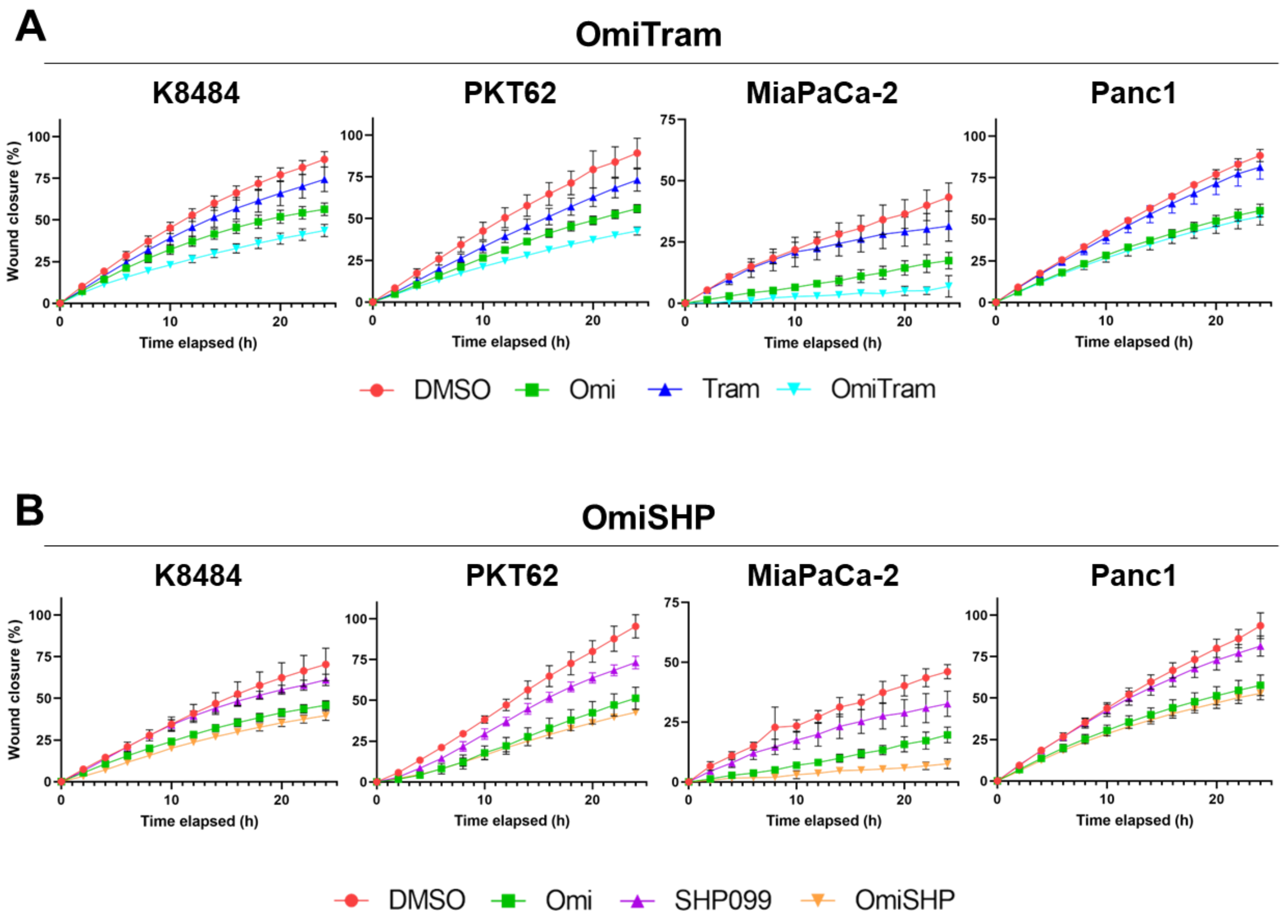

Supplemental Figure S3. Wound closure curves over 24 hours for each cell line and treatment condition in the migration assay shown in Figure 3. K8484 cells were treated with 5 nM Omipalisib, 5 nM Trametinib, and/or 50 µM of SHP099. PKT62 cells were treated with 10 nM Omipalisib, 10 nM Trametinib, and/or 50 µM of SHP099. MiaPaCa2 cells were treated with 25 nM Omipalisib, 20 nM Trametinib, and/or 20 µM SHP099. Panc1 cells were treated with 5 nM Omipalisib, 20 nM Trametinib, and/or 20 µM SHP099. n=4-6 wells for all groups. Error bars are shown ± SD.

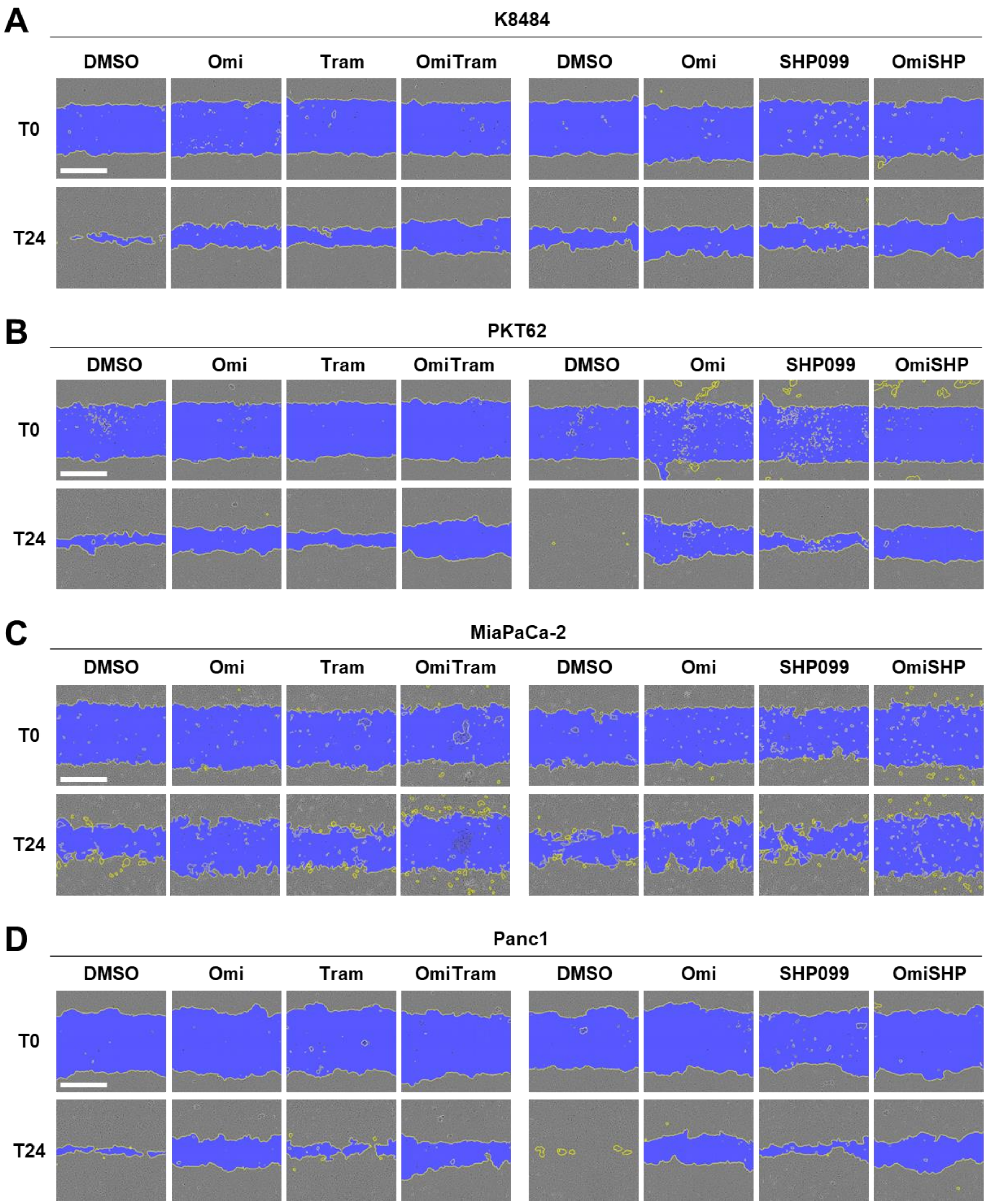

Supplemental Figure S4. Representative images of wound closure at T0 and T24 for each cell line and treatment condition as quantified in Figure 3. K8484 cells were treated with 5 nM Omipalisib, 5 nM Trametinib,

and/or 50 µM of SHP099. PKT62 cells were treated with 10 nM Omipalisib, 10 nM Trametinib, and/or 50 µM of SHP099. MiaPaCa2 cells were treated with 25 nM Omipalisib, 20 nM Trametinib, and/or 20 µM SHP099. Panc1 cells were treated with 5 nM Omipalisib, 20 nM Trametinib, and/or 20 µM SHP099. (Scale bar=600 µm).

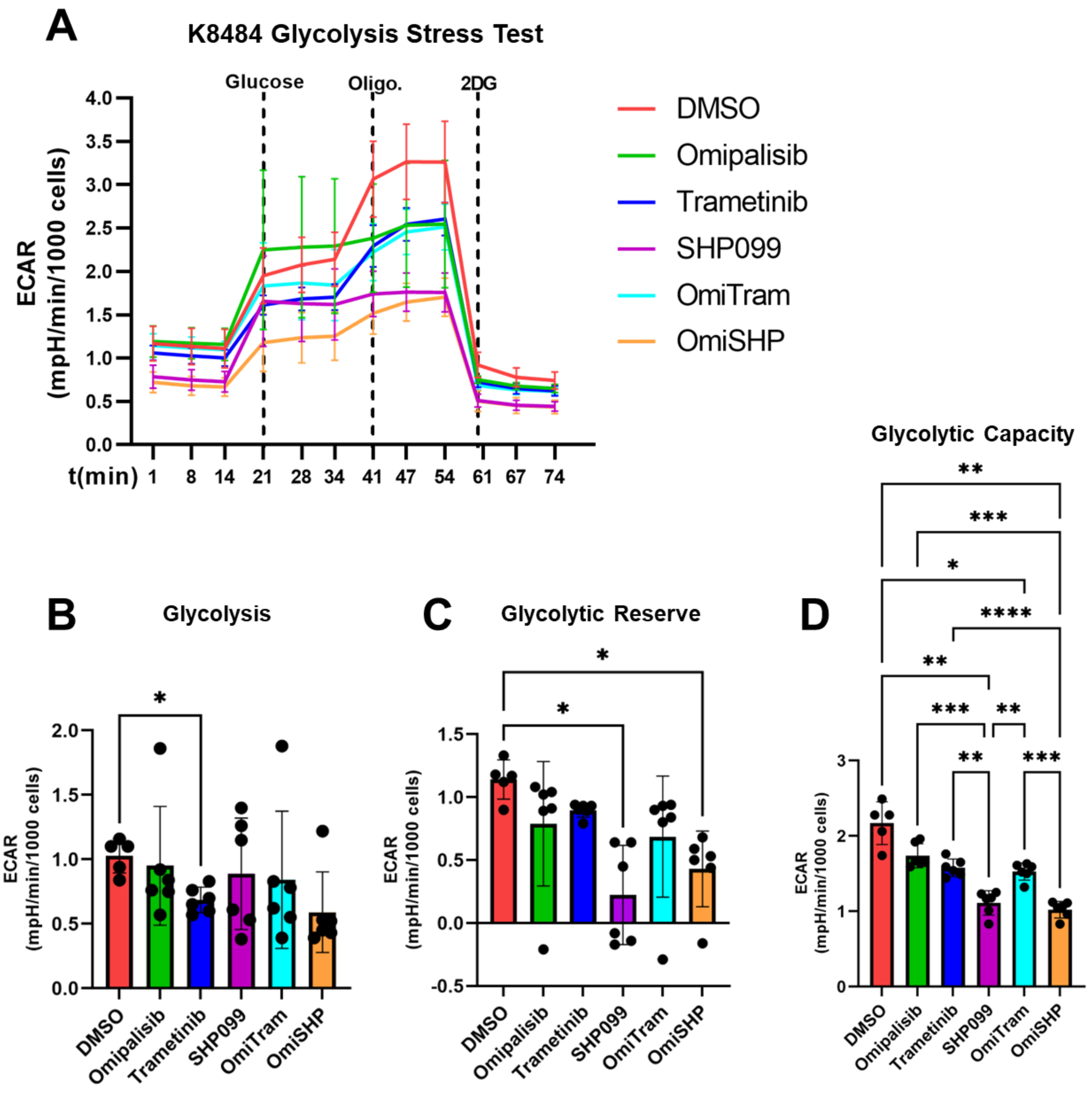

Supplemental Figure S5. Combined targeting of PI3K and MAPK pathways suppresses glycolytic capacity in K8484 cells in vitro. (A-D) Extracellular acidification rate (ECAR) measured using Agilent Seahorse Glycolysis Stress in a Seahorse XFe96 bioanalyzer normalized to cell count per well. Basal ECAR was measured prior to subsequent injections of glucose (10mM final conc.), Oligomycin (1µM final conc.), and 2-DG (100mM final conc.). (B) Glycolysis rates of treated cells, measured by subtracting basal ECAR from the maximal value following glucose injection. (C) Glycolytic reserve of treated cells, measured by subtracting the maximal ECAR

following Oligomycin injection from the maximal value following glucose injection. (D) Glycolytic capacity, measured by subtracting minimum ECAR following 2-DG injection from the maximal ECAR following Oligomycin injection. Cells were treated with 25 nM (single agent or OmiTram) or 10nM (OmiSHP) Omipalisib, 10 nM Trametinib, and/or 20 μM SHP099. Vehicle: n=5; Omipalisib: n=6; Trametinib: n=6; SHP099: n=6; OmiTram: n=6; OmiSHP n=6. (*$p < 0.05$, **$p < 0.01$, ***$p < 0.001$, and ****$p < 0.0001$). Error bars are shown ± SD.

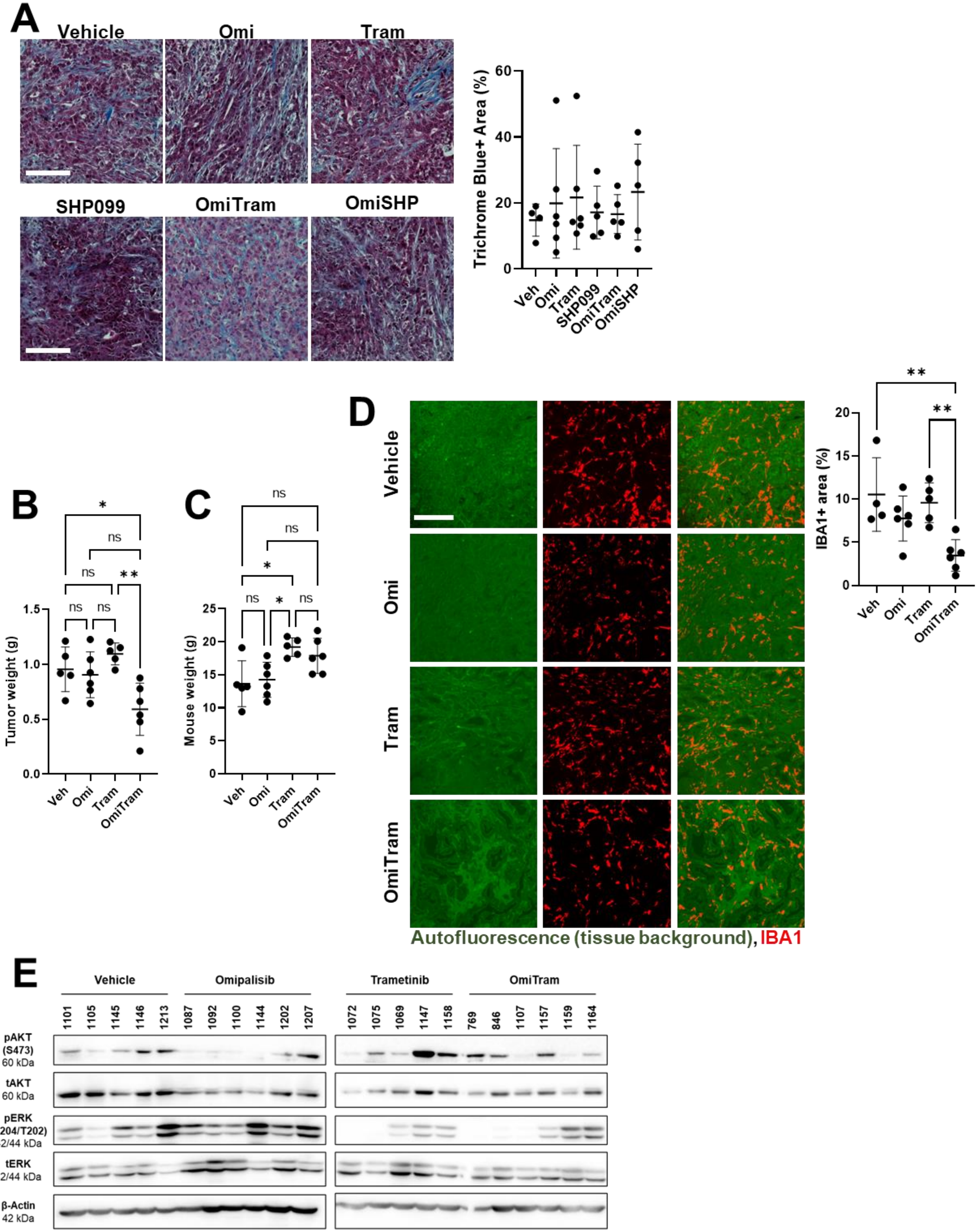
A
Vehicle
Omi
Tram
SHP099
OmiTram
OmiSHP
Trichrome Blue+ Area (%)
0
20
40
60
Veh
Omi
Tram
SHP099
OmiTram
OmiSHP
B
Tumor weight (g)
0.0
0.5
1.0
1.5
*
ns
ns
**
ns
ns
Veh
Omi
Tram
OmiTram
C
Mouse weight (g)
0
5
10
15
20
25
ns
ns
*
ns
*
ns
Veh
Omi
Tram
OmiTram
D
Vehicle
Omi
Tram
OmiTram
Autofluorescence (tissue background), IBA1
IBA1+ area (%)
0
5
10
15
20
**
**
Veh
Omi
Tram
OmiTram
E
Vehicle
Omipalisib
Trametinib
OmiTram
1101
1105
1145
1146
1213
1087
1092
1100
1144
1202
1207
1072
1075
1069
1147
1158
769
846
1107
1157
1159
1164
pAKT (S473) 60 kDa
tAKT 60 kDa
pERK (Y204/T202) 42/44 kDa
tERK 42/44 kDa
β-Actin 42 kDa

Supplemental Figure S6. Additional *in vivo* tumor analysis. (A) Representative 20x images of Trichrome staining for day 18 tumors from the subcutaneous implantation experiment described in Figure 4. Quantification represents average percent of blue area per 20x field of view in cell-rich, non-necrotic areas (n=2-3 fields of view). Subcutaneous tumor analysis: vehicle: n=4; Omipalisib: n=6; Trametinib n=6; SHP099: n=5; OmiTram: n=5; OmiSHP: n=5. (ns = not significant ($p \geq 0.05$)). Error bars are shown ± SD. (B,C) Endpoint tumor weight (g) and mouse weight (g) from PKT mice in Figure 5. (D) Representative 20x images of IBA1 staining (red). Green autofluorescence is included to show tissue background behind IBA1 stain. Quantification represents average percent IBA1+ area per 20x field of view (n=3 random fields of view). PKT post-mortem analysis: vehicle: n=4 (one tissue excluded due to technical error); Omipalisib: n=6; Trametinib: n=5; OmiTram n=6. (*$p < 0.05$, **$p < 0.01$). Scale bars=125 μm. Error bars are shown ± SD. (E) Western blot examination of pathway activation markers in endpoint PKT tumors (Figure 5).

K8484 OmiTram Group (Figure 1)

File S1. Annotated uncropped images of Western blots with markers.

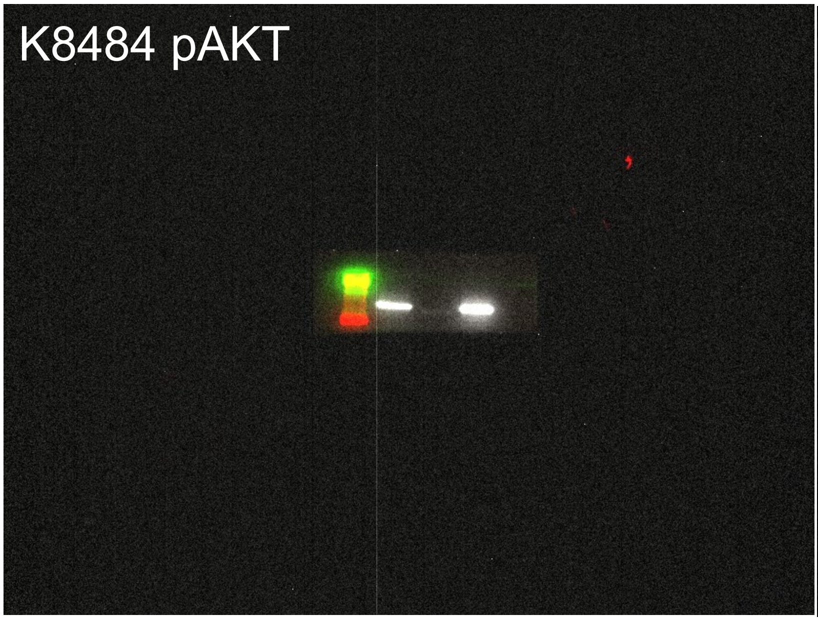

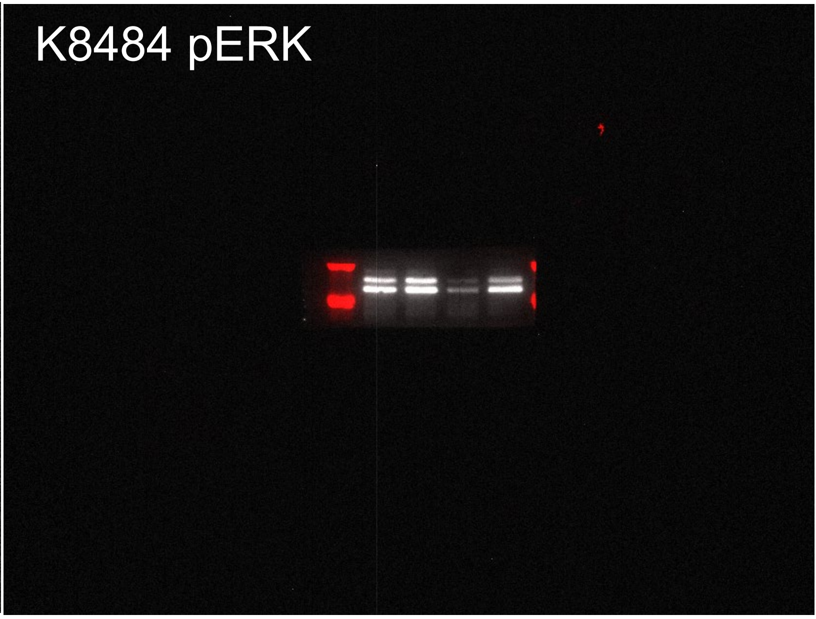

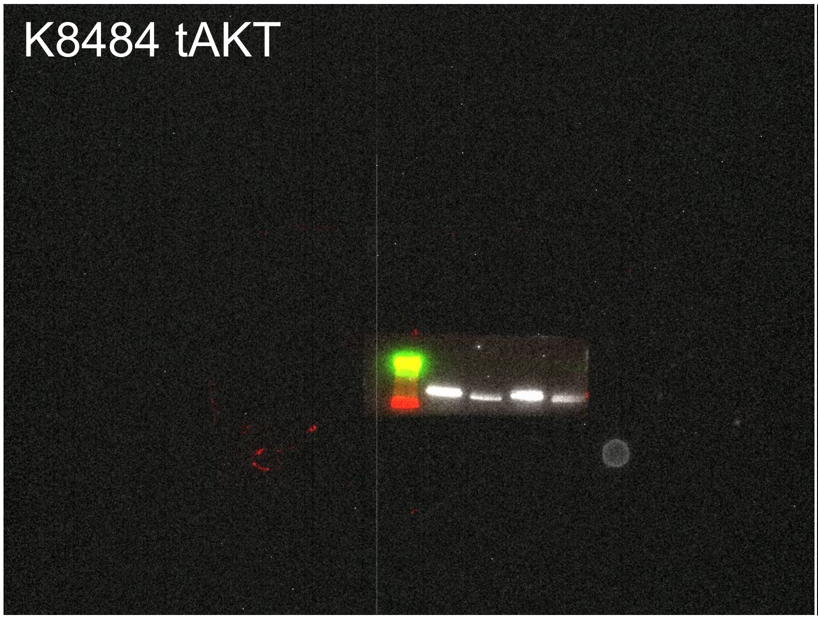

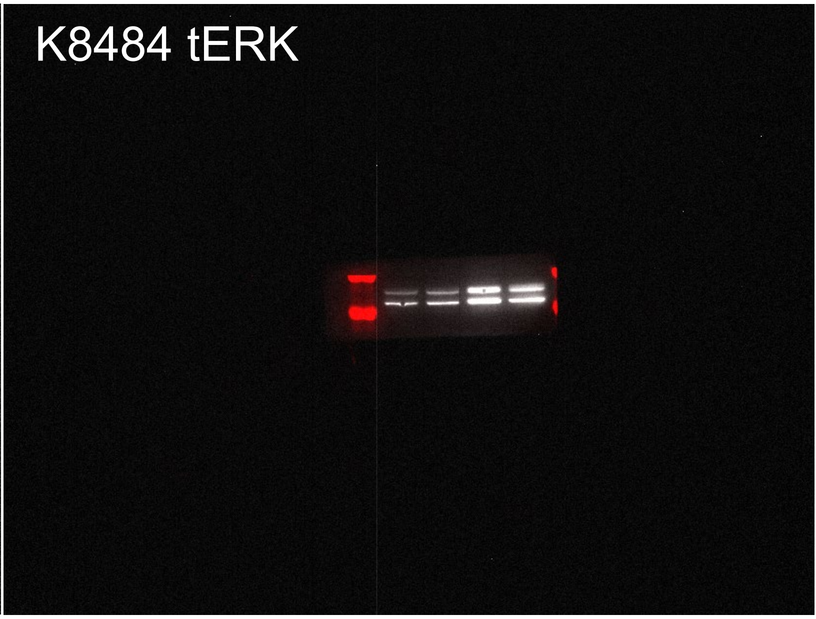

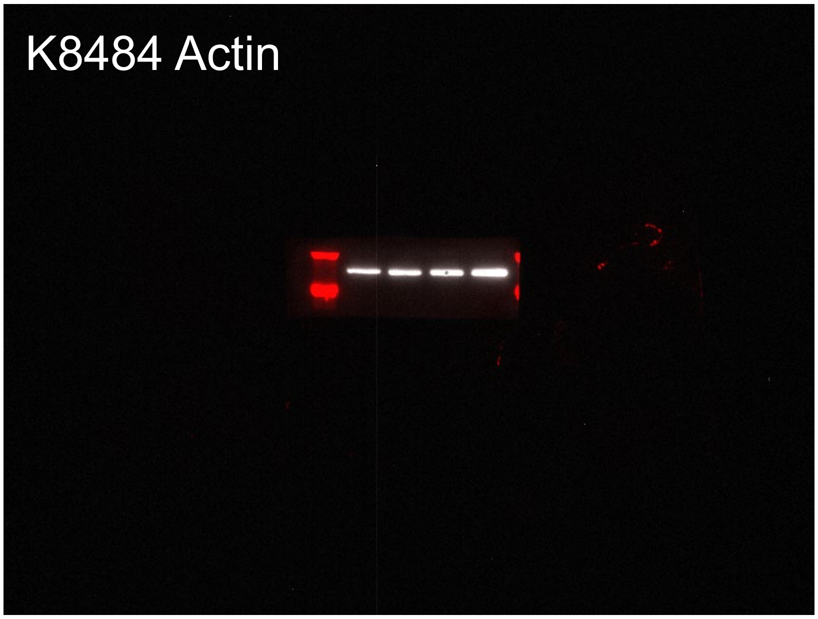

K8484 OmiSHP Group (Figure 1)

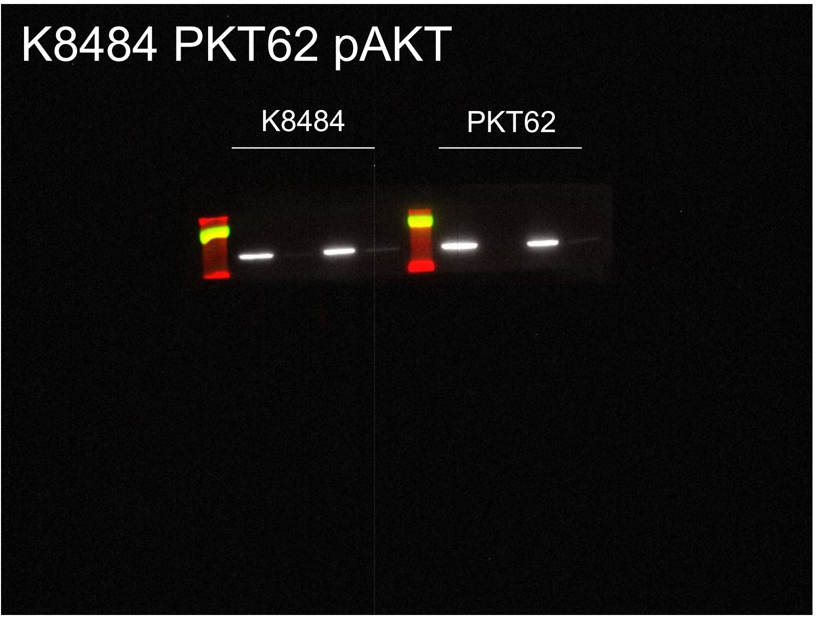

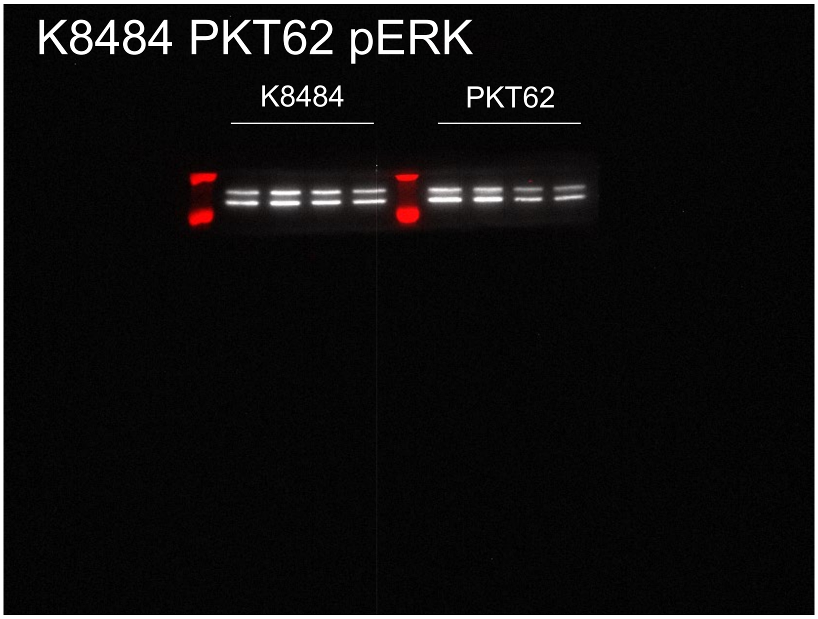

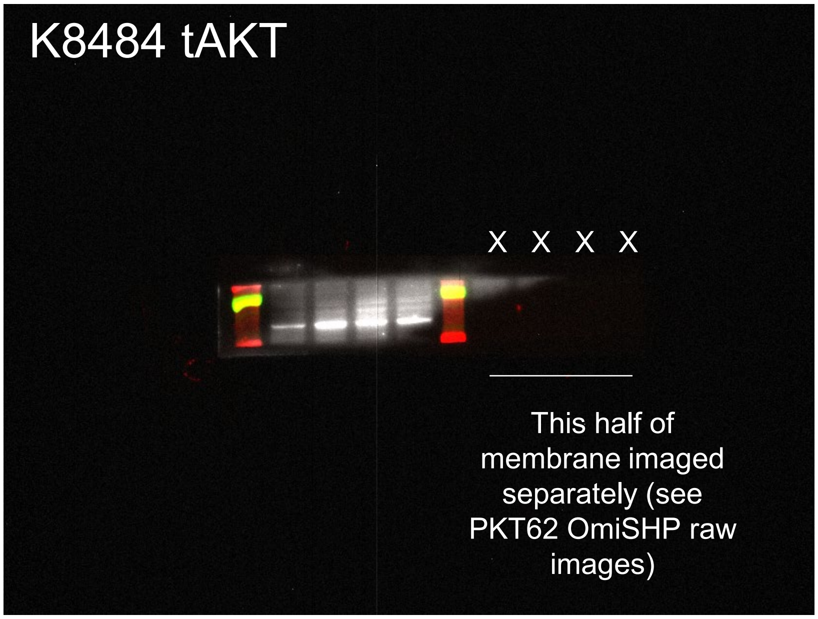

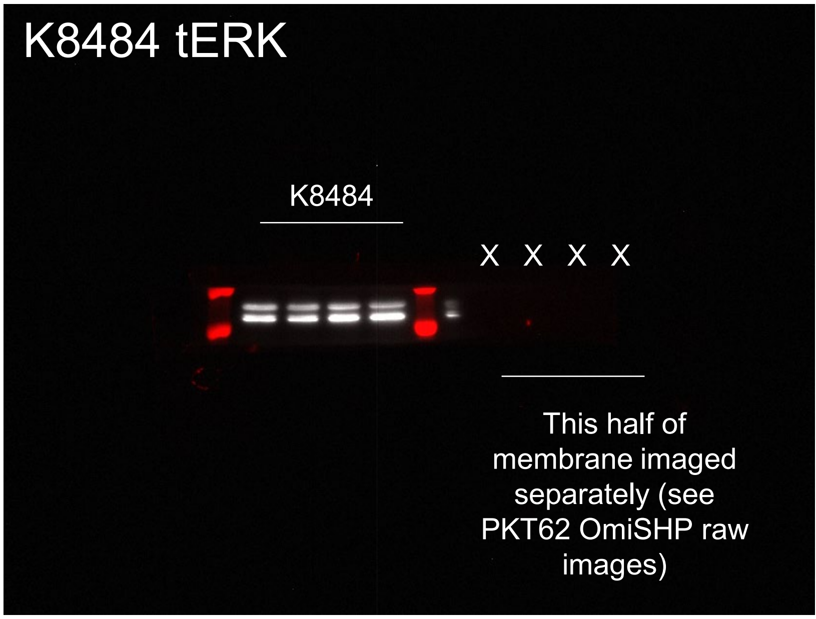

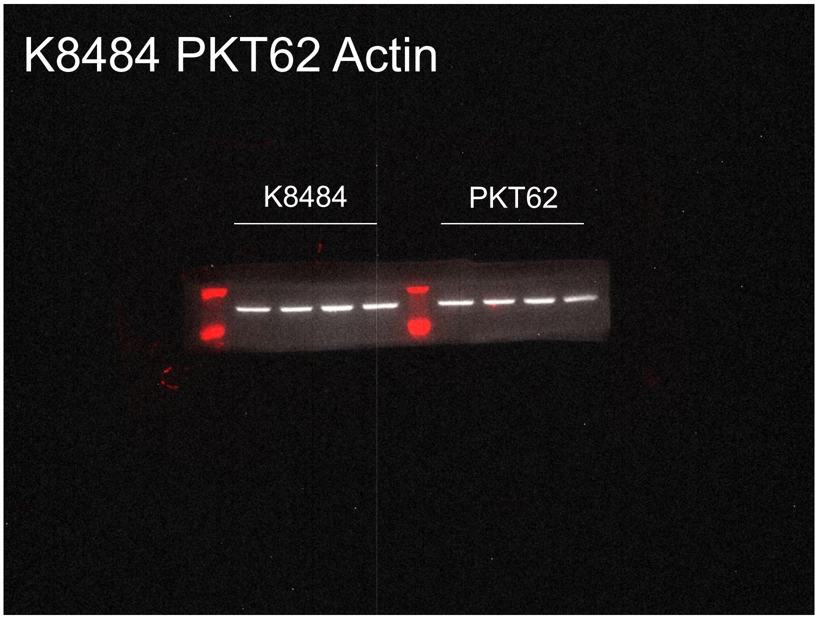

PKT62 OmiTram Group (Figure 1)

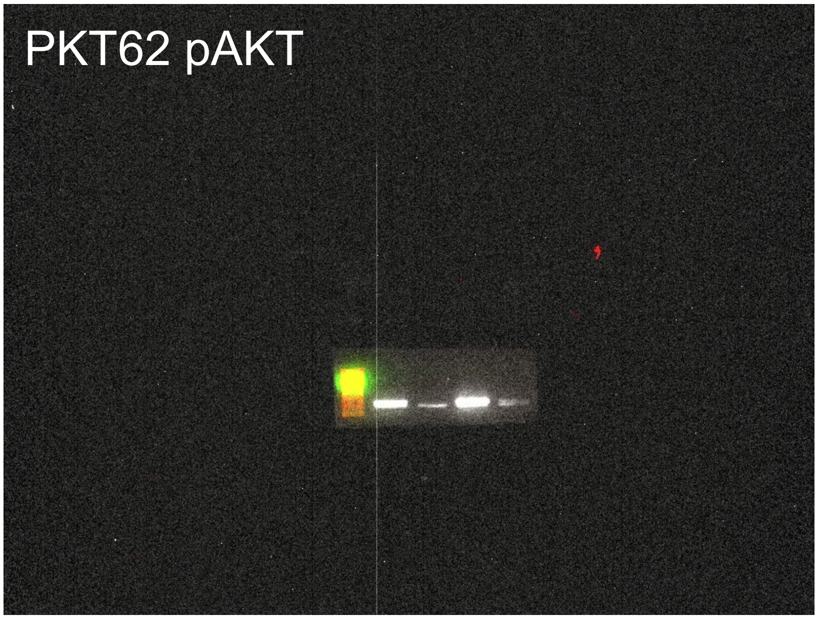

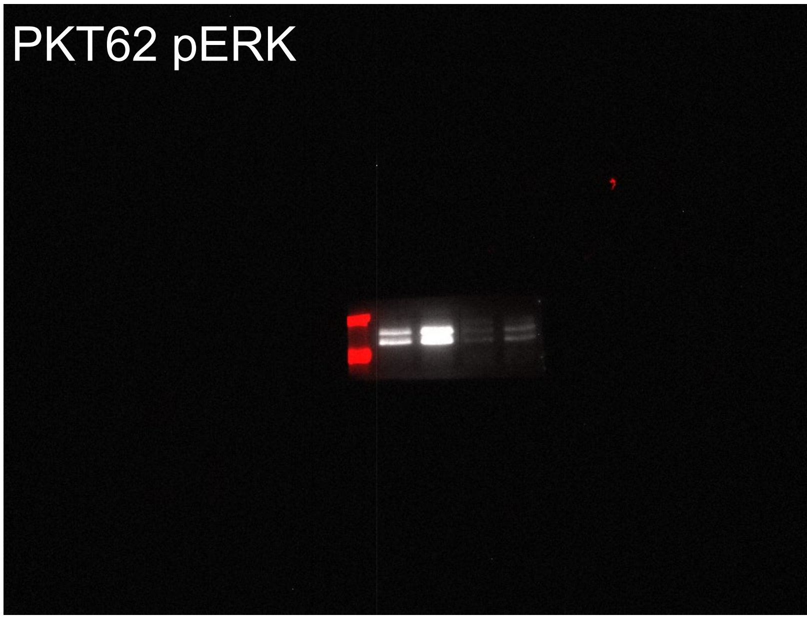

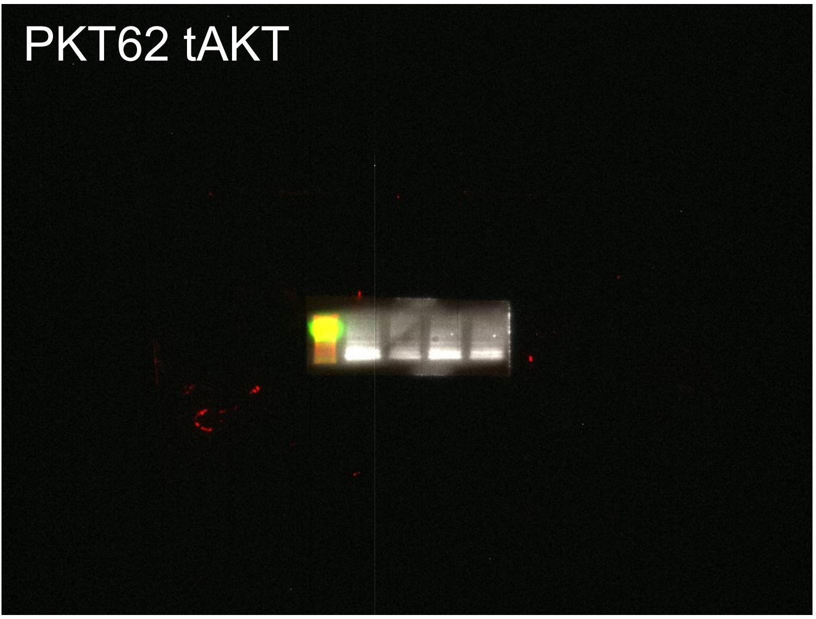

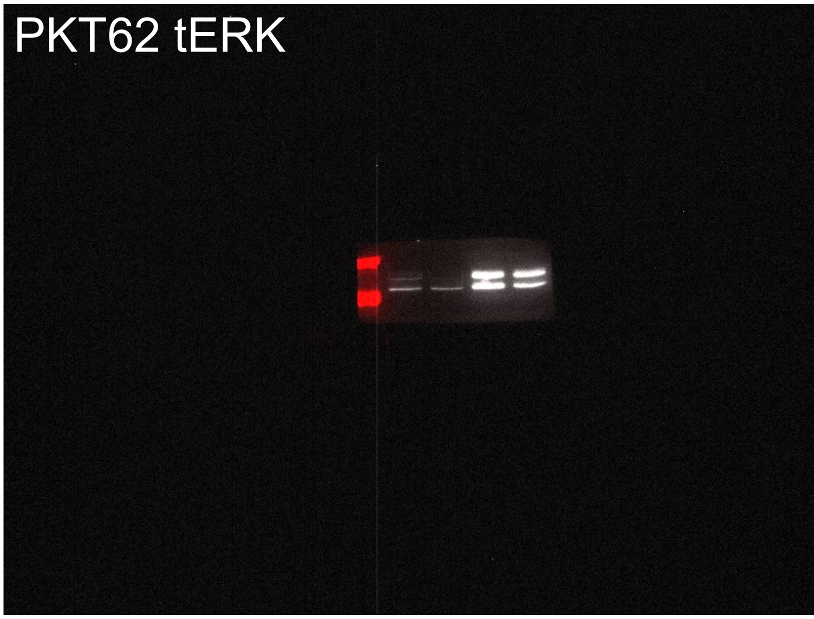

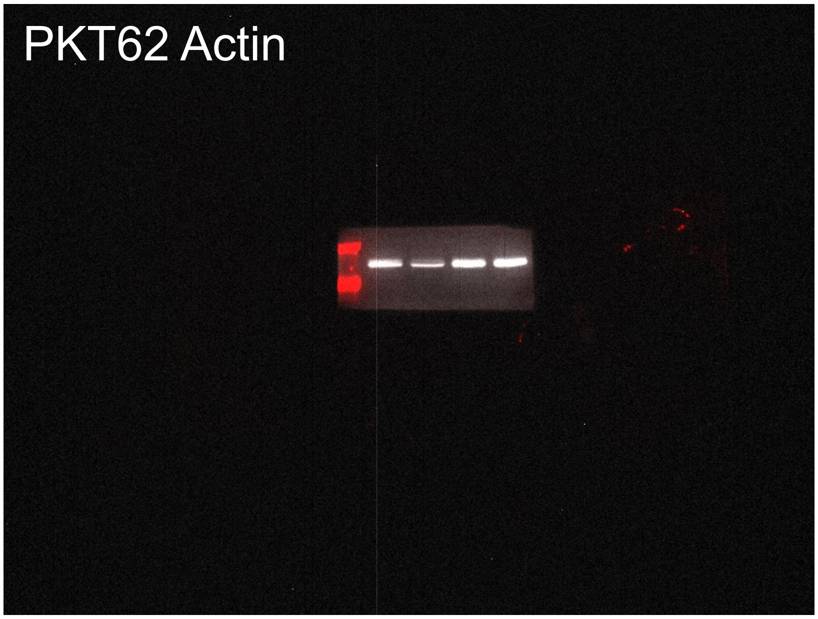

PKT62 OmiSHP Group (Figure 1)

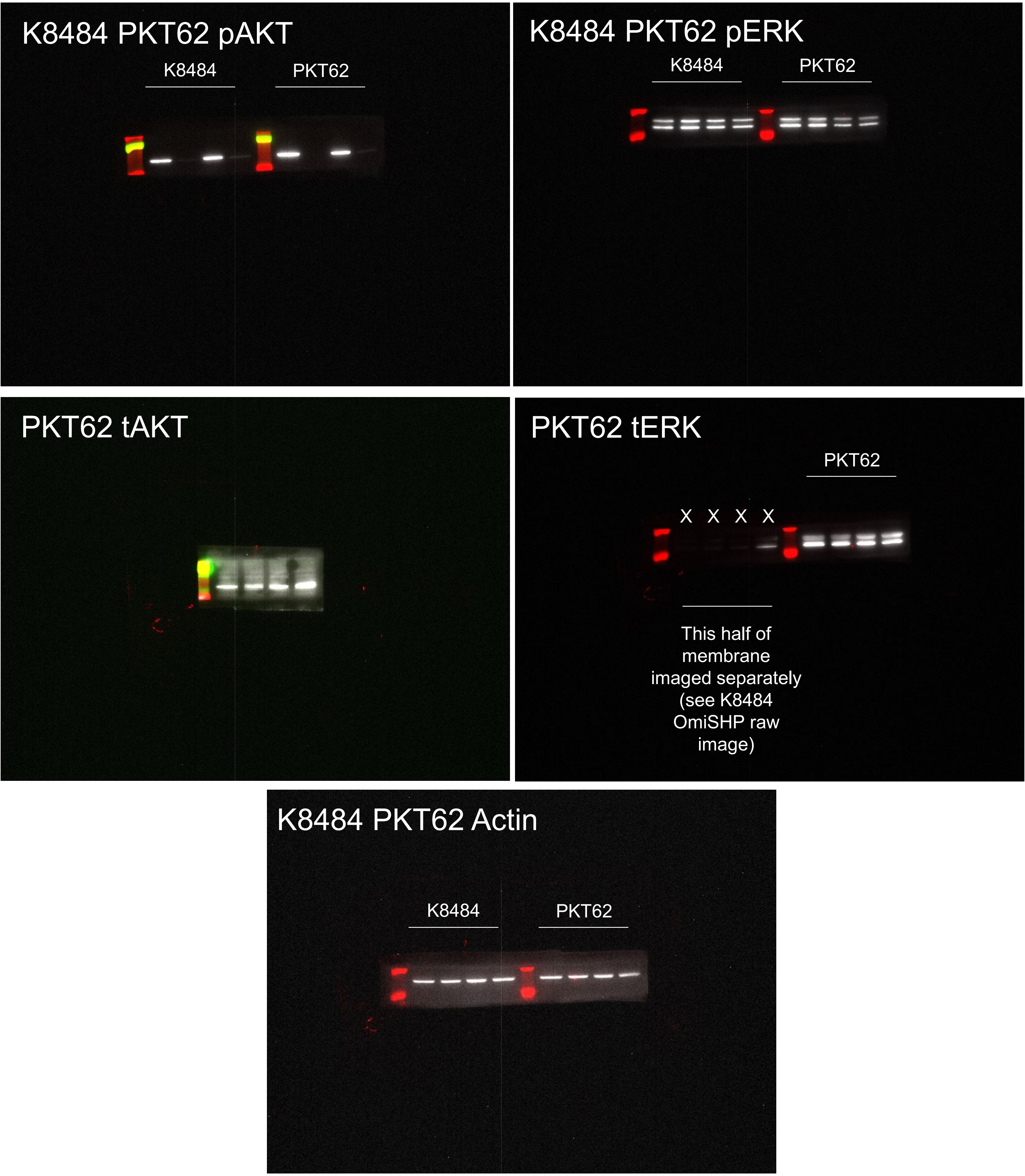

MiaPaCa-2 OmiTram Group (Figure 1)

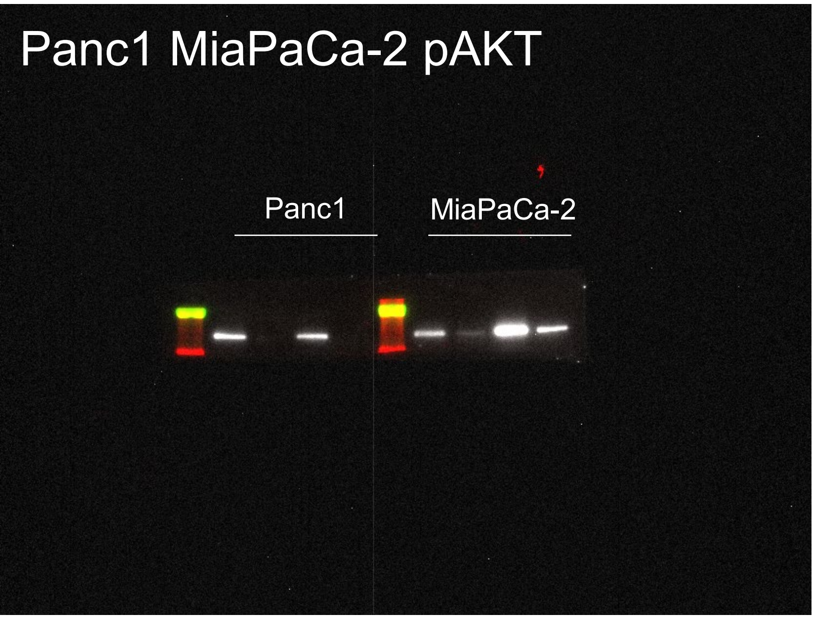

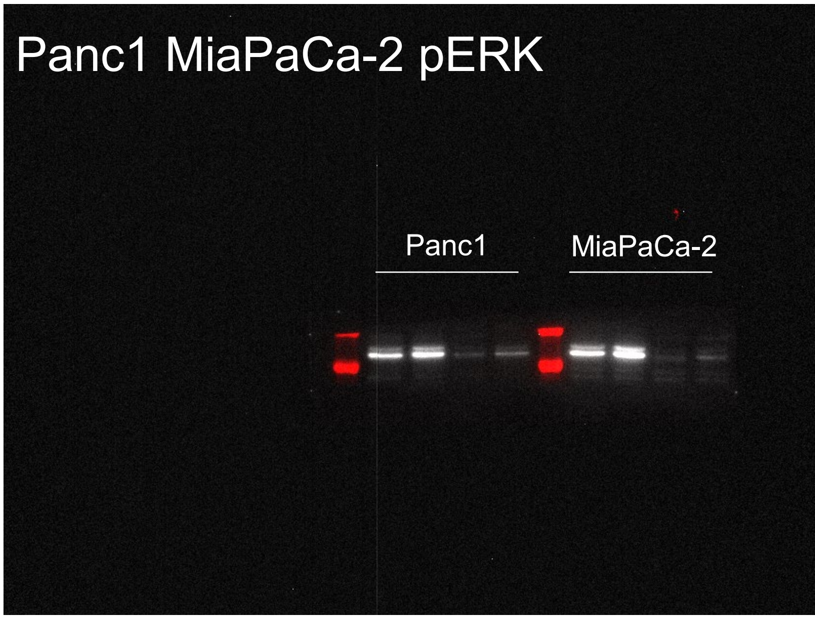

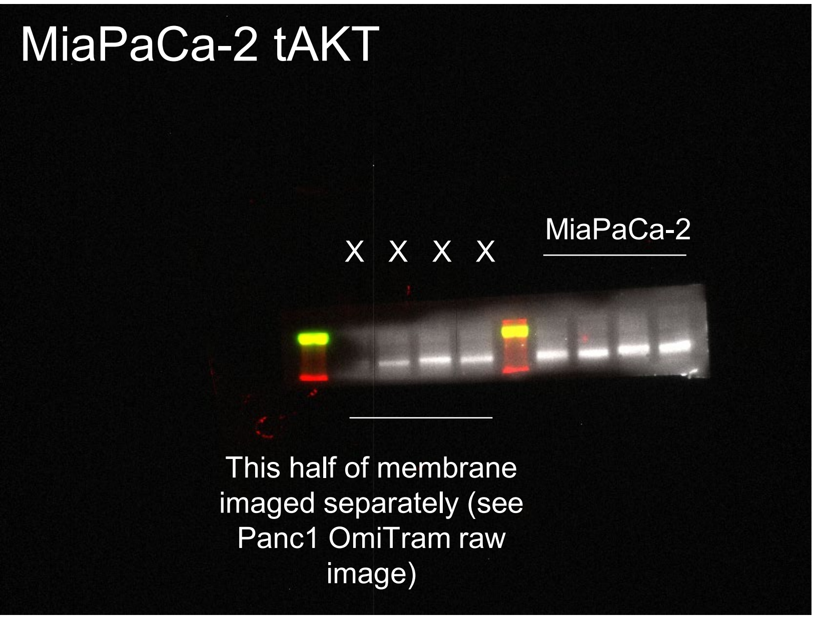

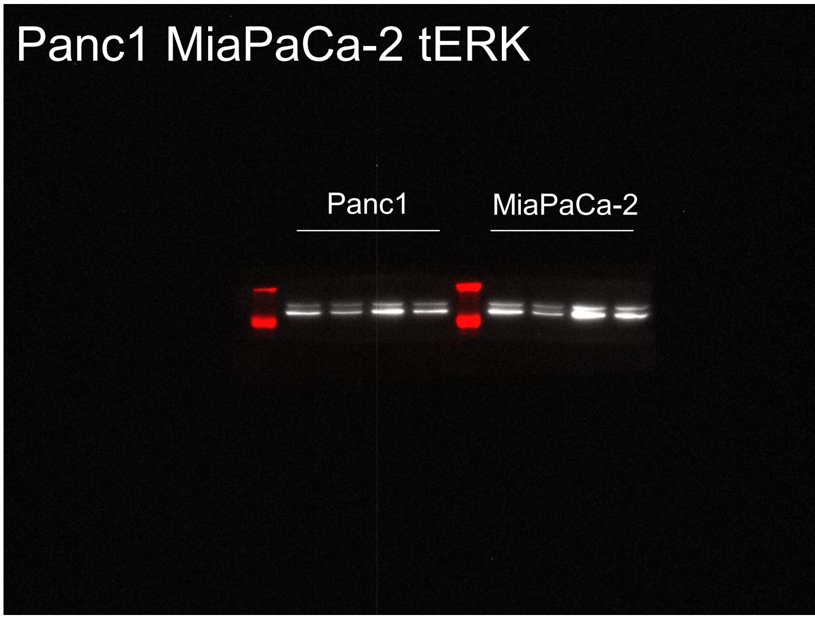

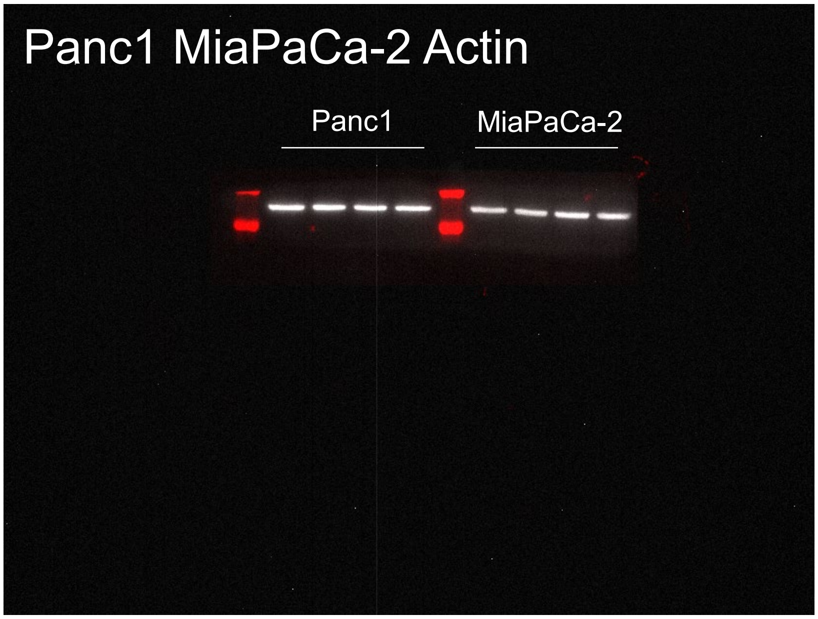

MiaPaCa-2 OmiSHP Group (Figure 1)

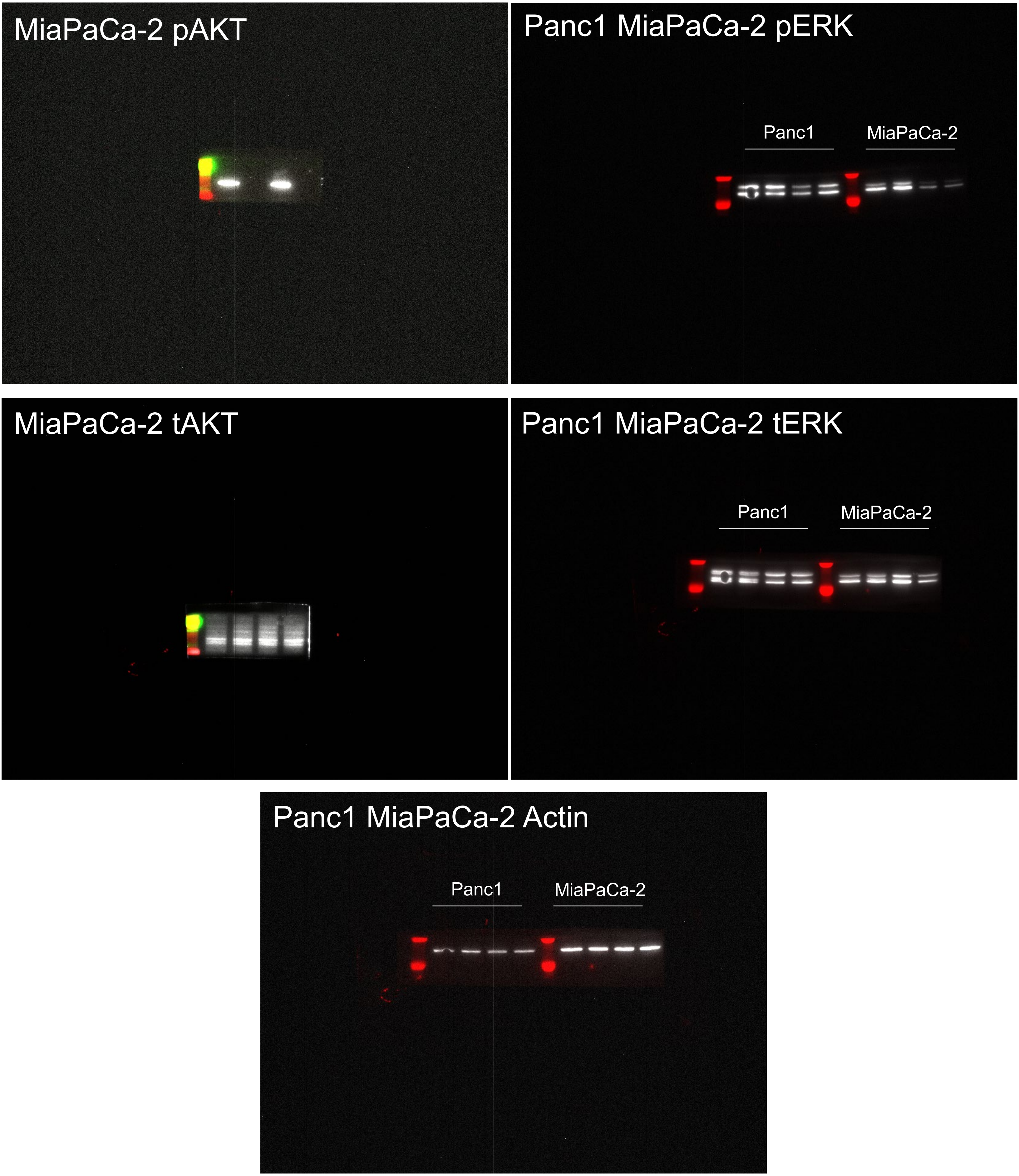

Panc1 OmiTram Group (Figure 1)

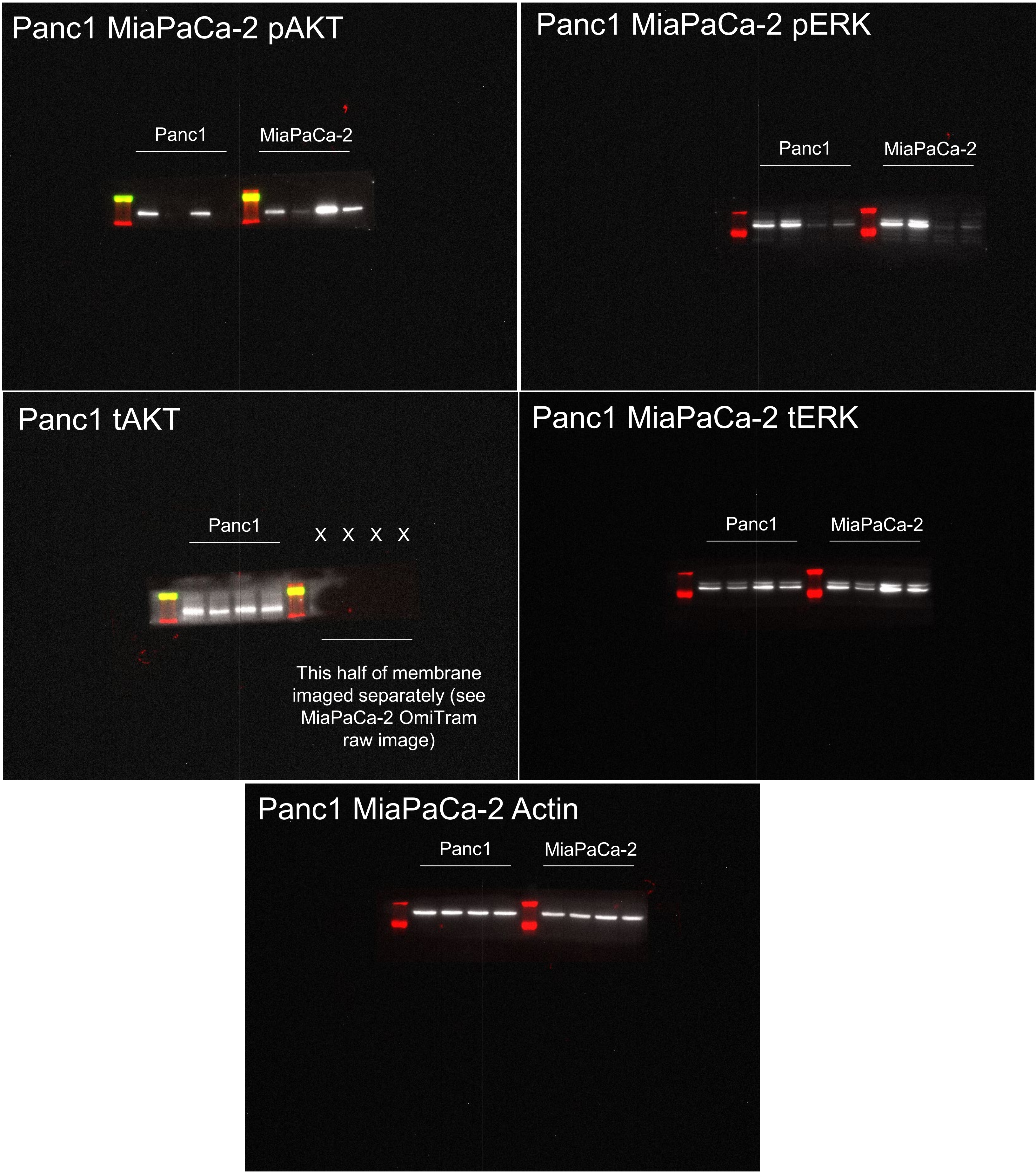

Panc1 OmiSHP Group (Figure 1)

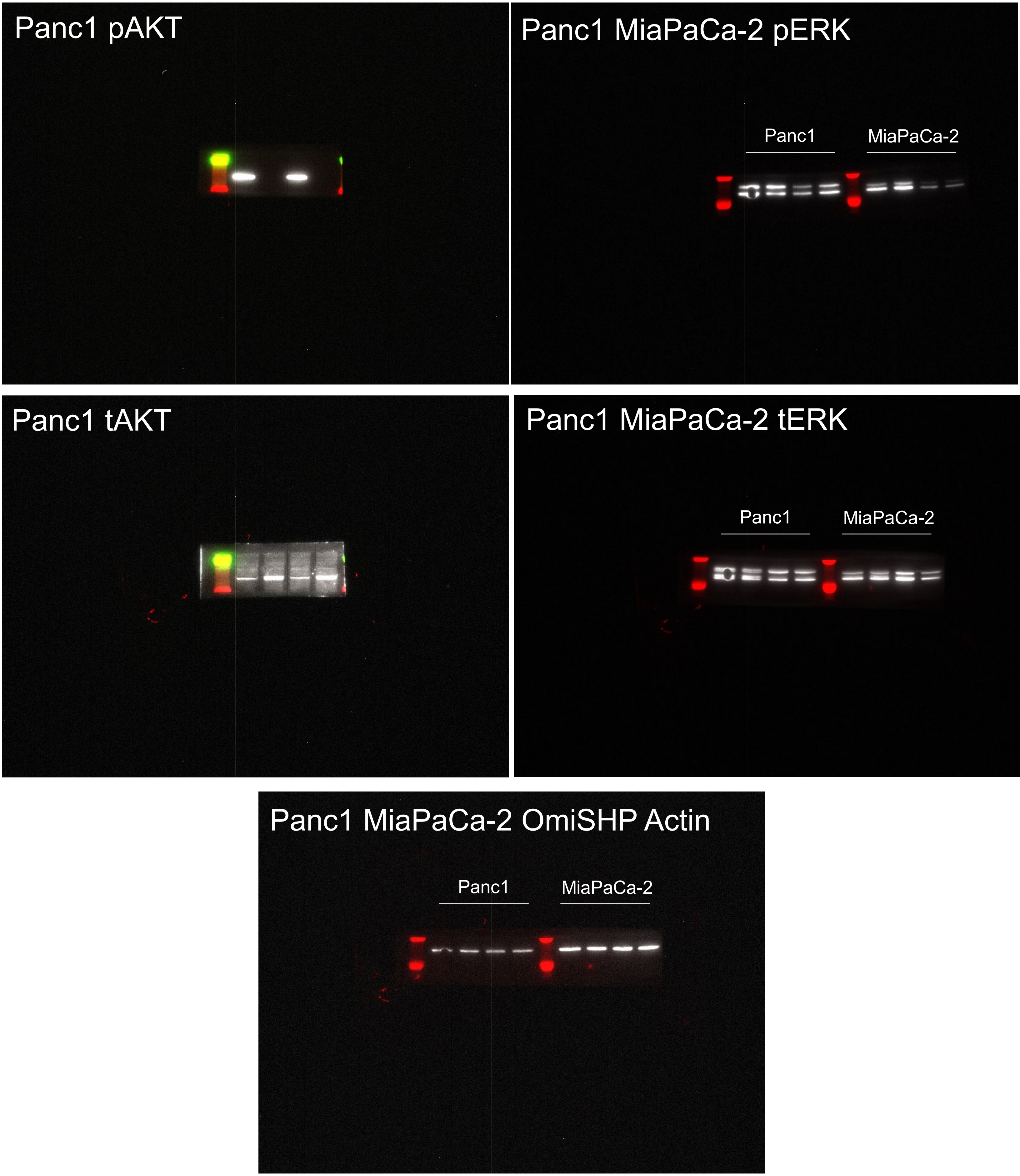

K8484 Omipalisib Dose Response (Figure S1)

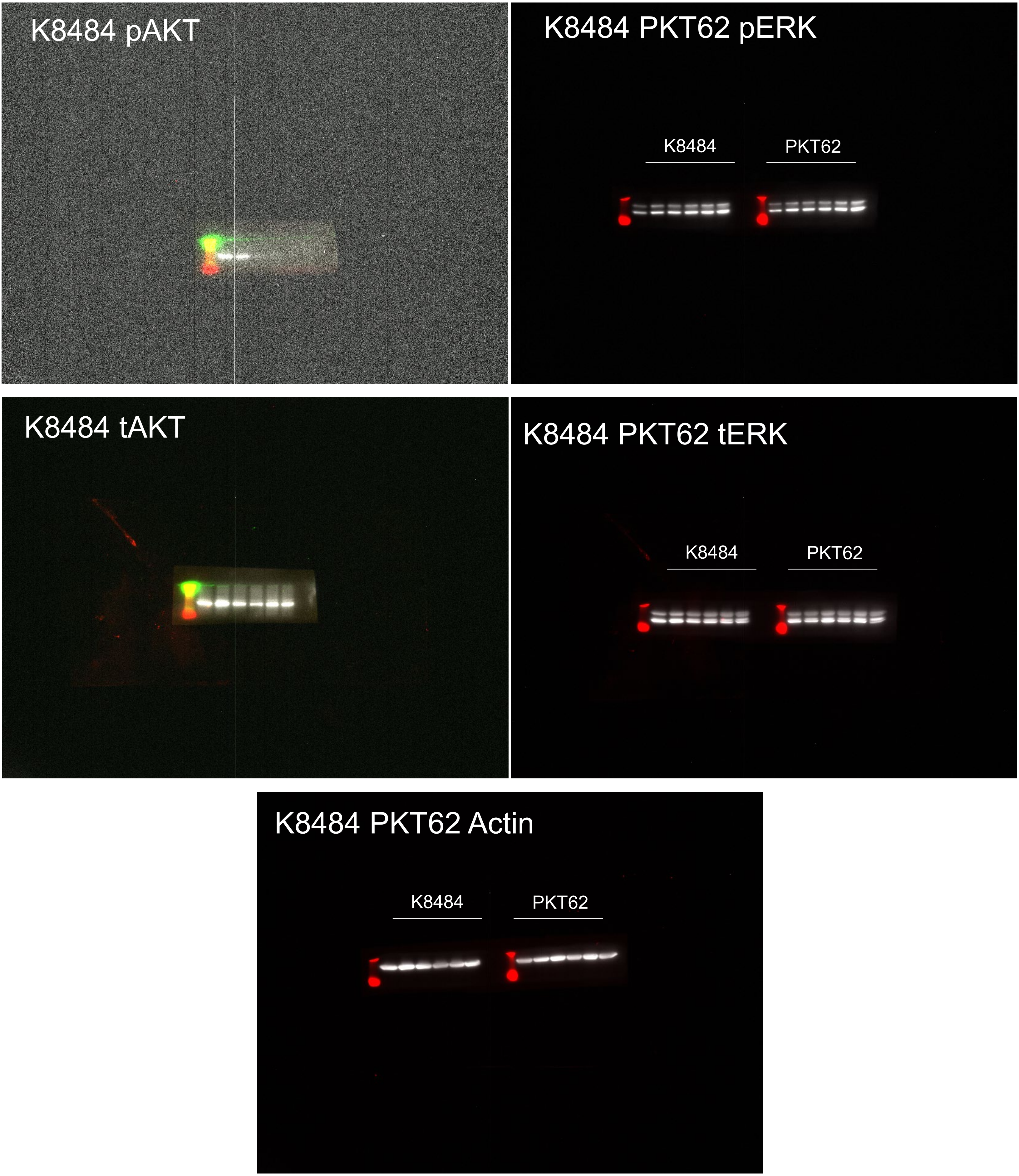

PKT62 Omipalisib Dose Response (Figure S1)

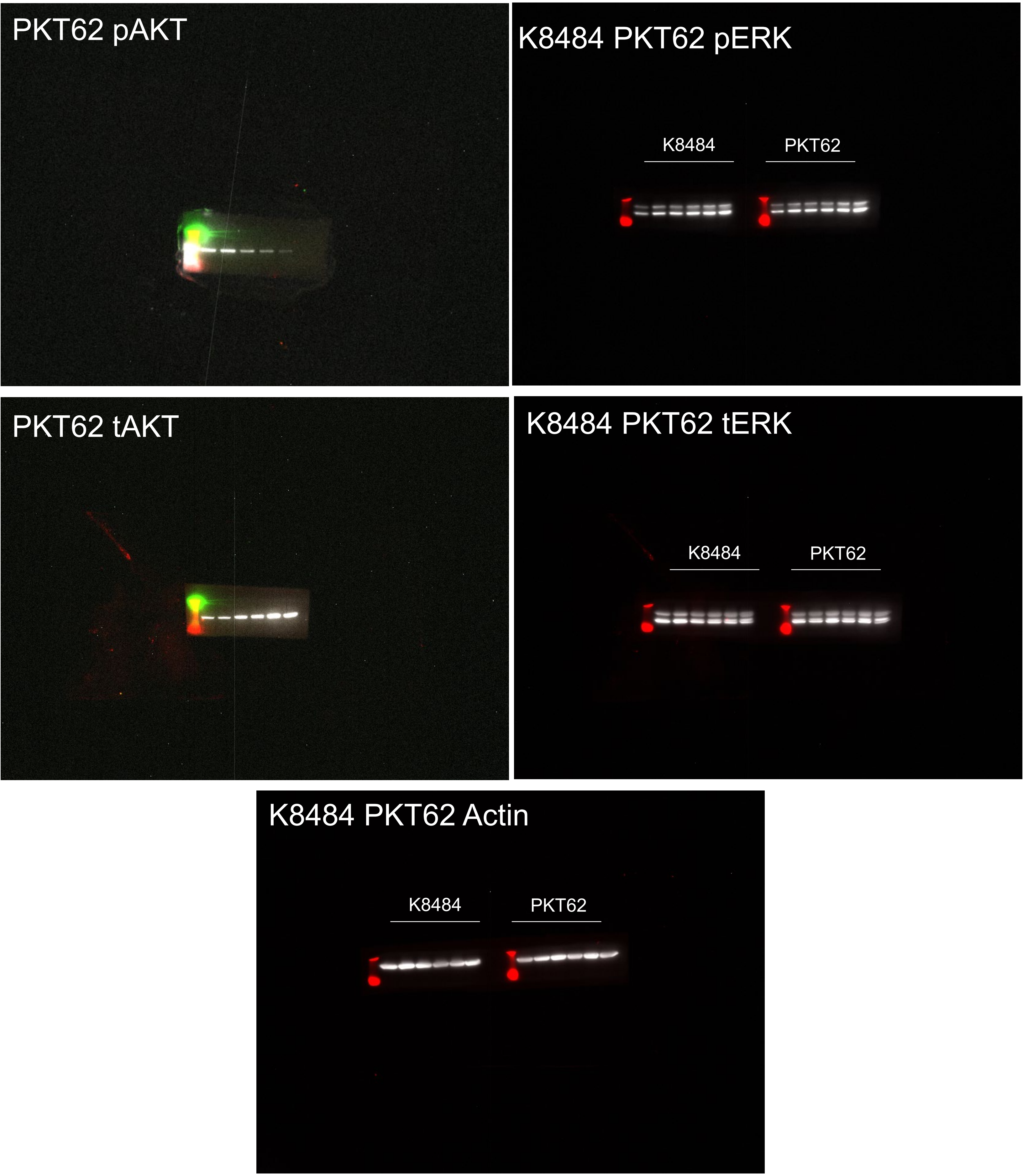

MiaPaCa-2 Omipalisib Dose Response (Figure S1)

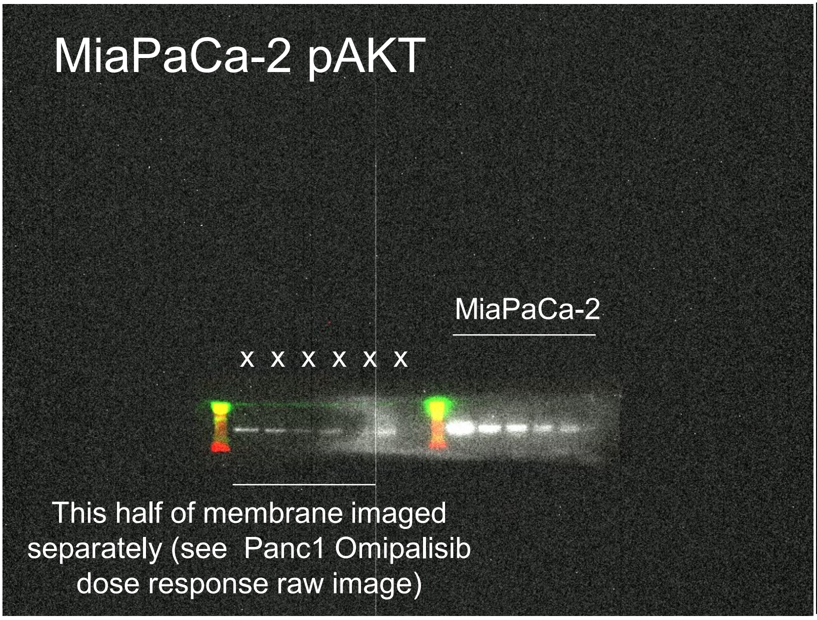

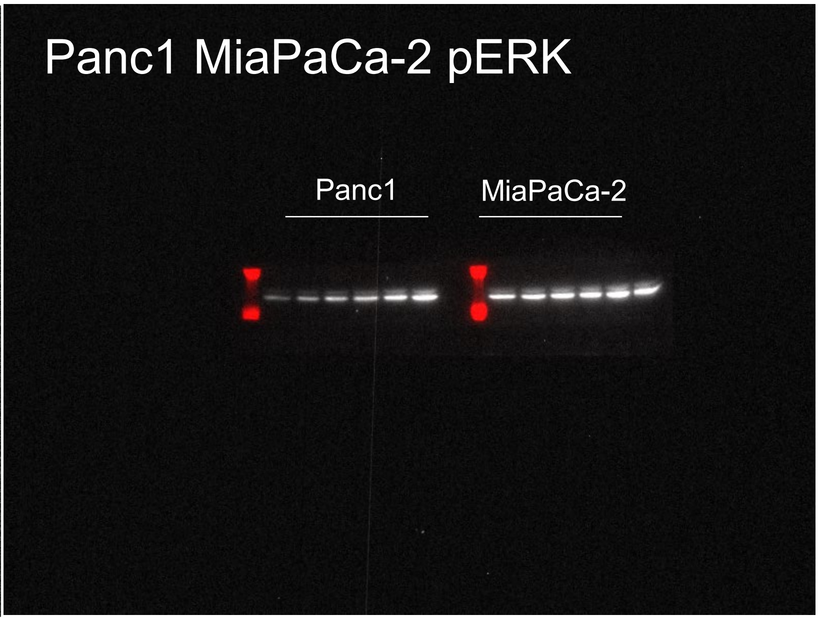

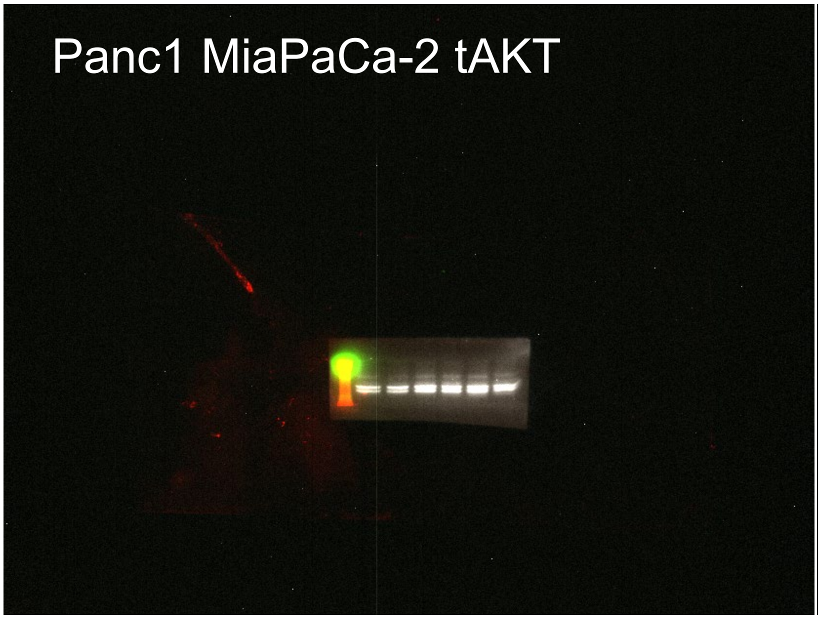

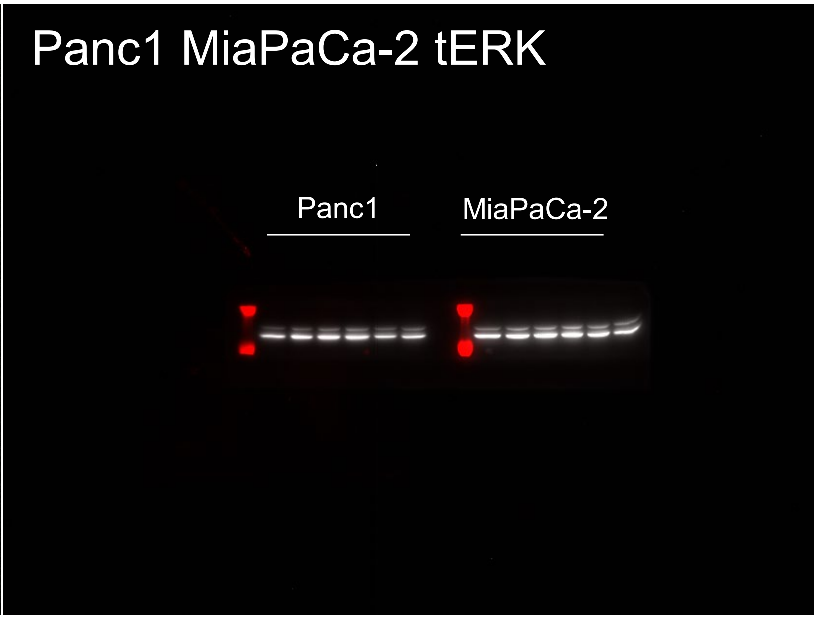

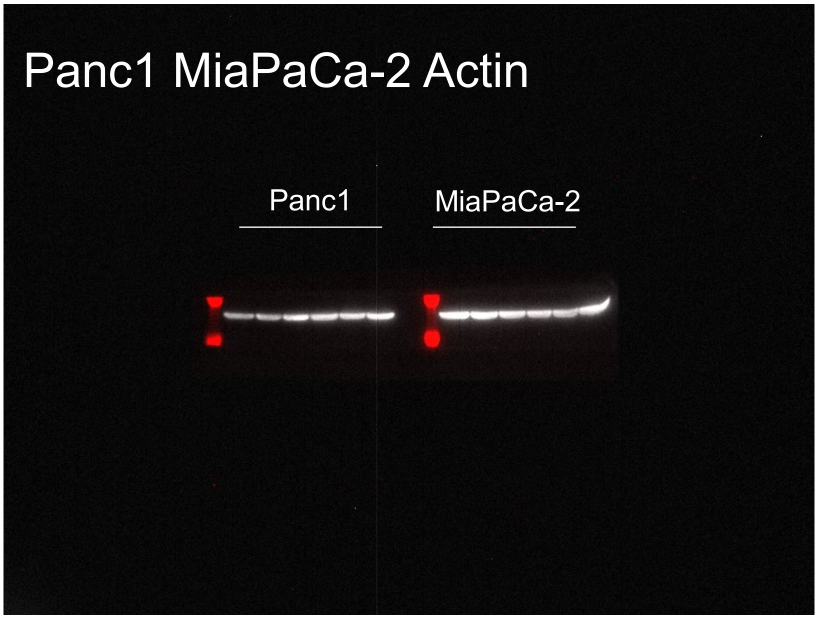

Panc1 Omipalisib Dose Response (Figure S1)

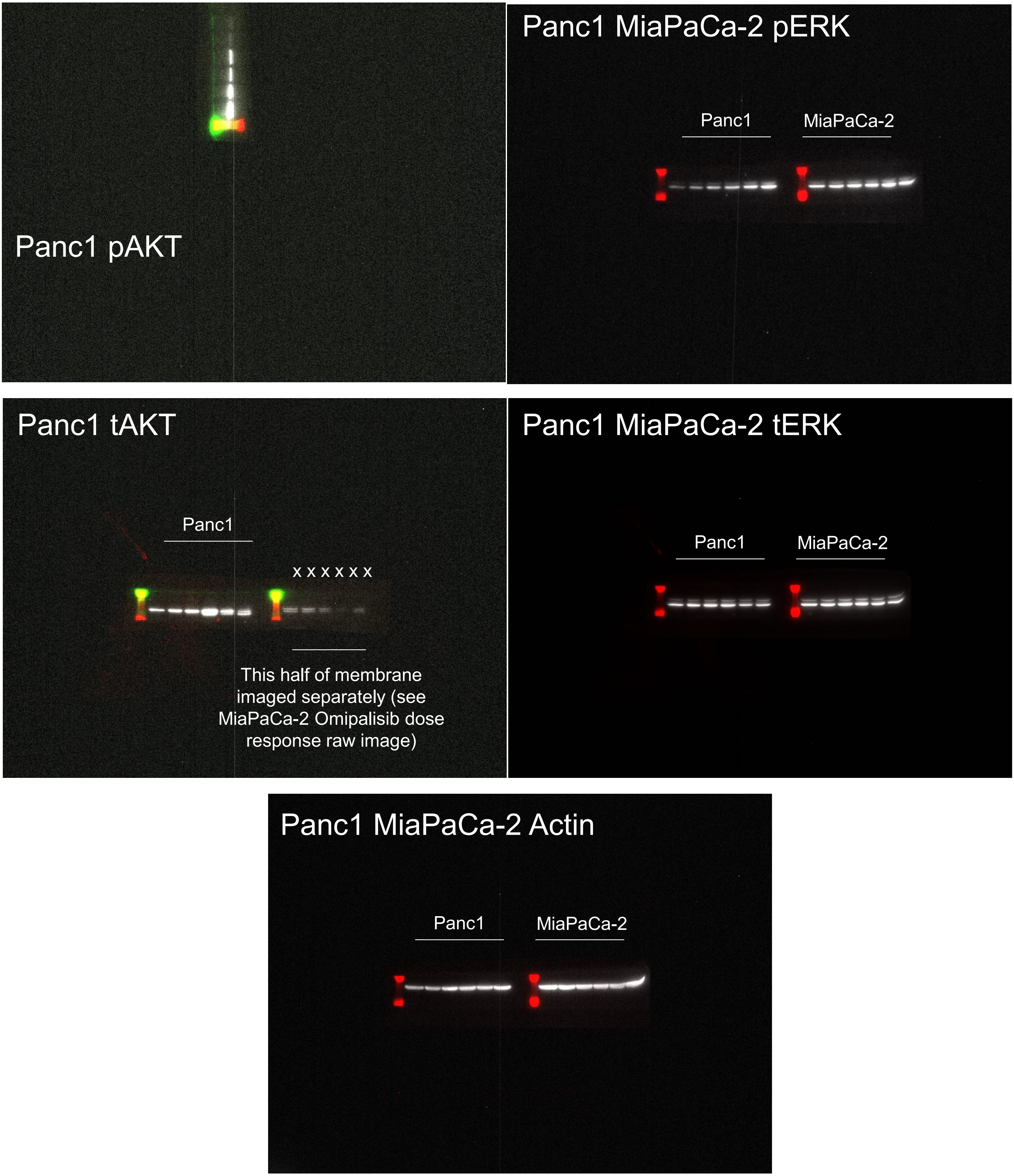

K8484 Trametinib Dose Response (Figure S1)

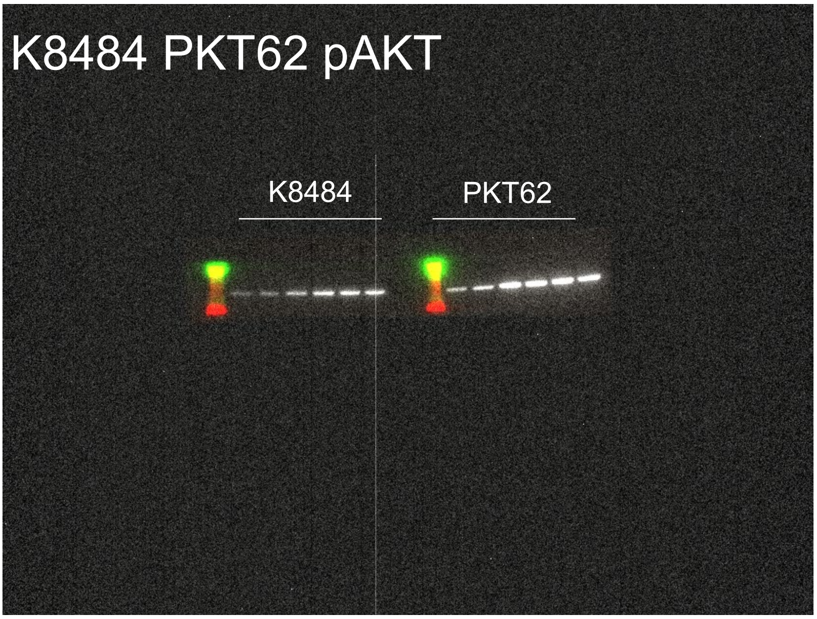

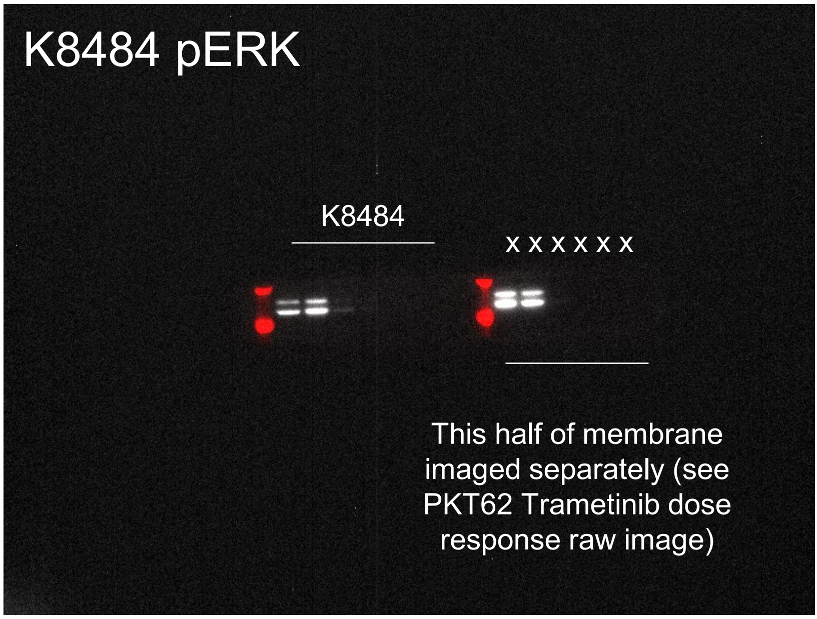

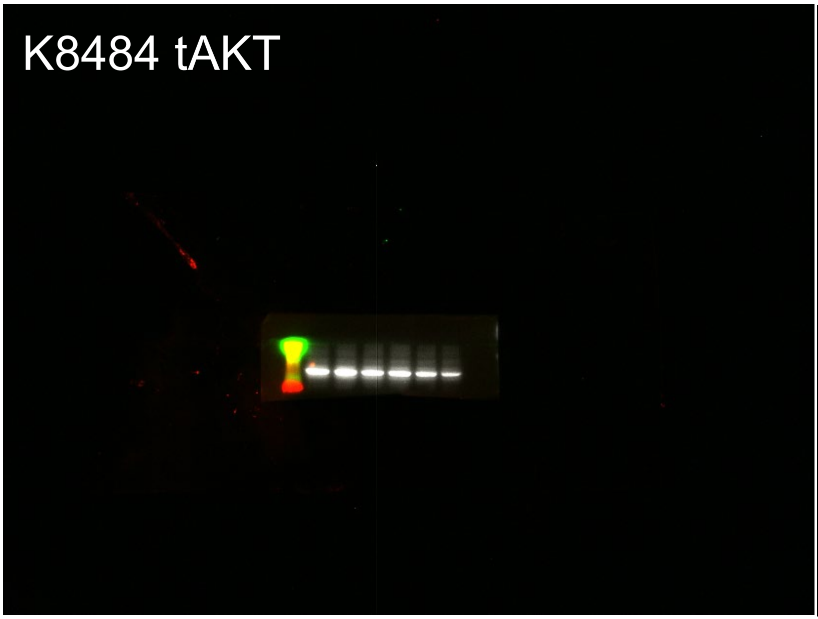

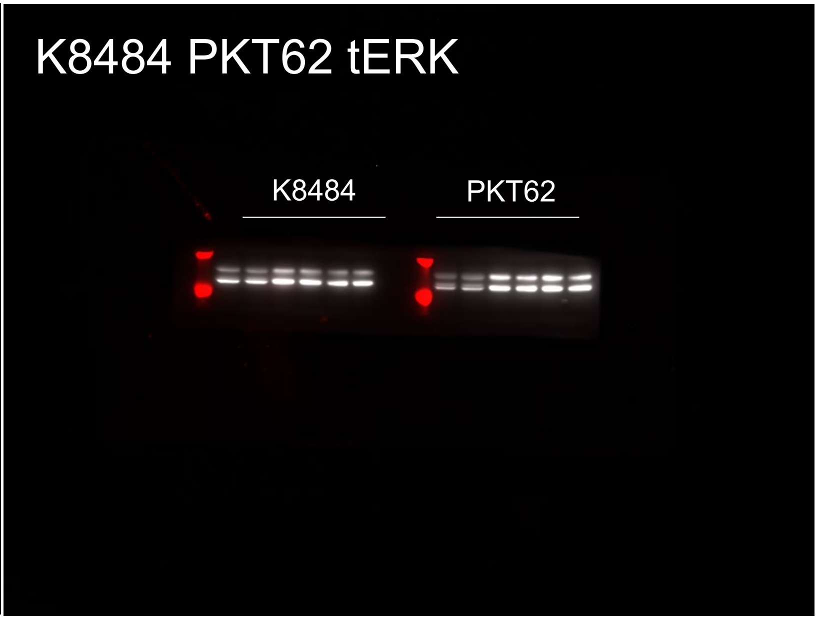

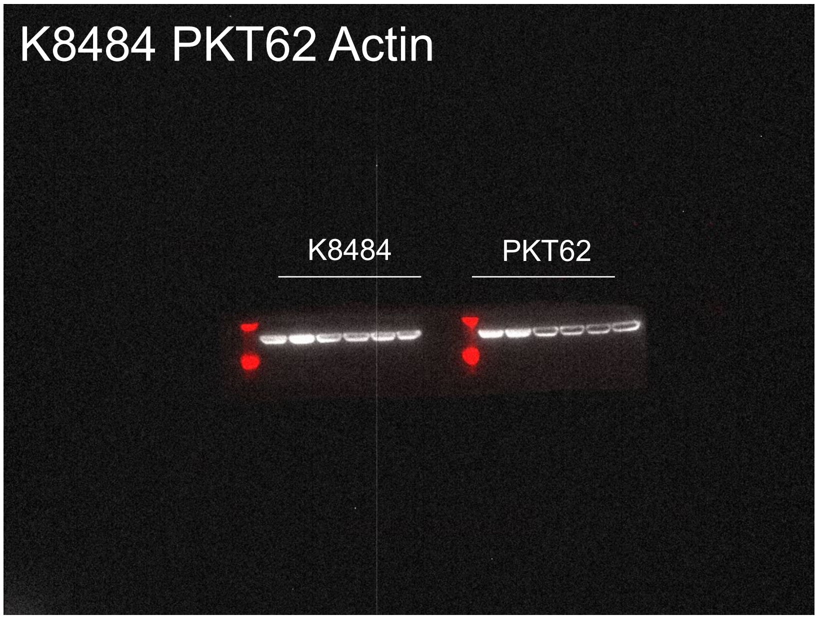

PKT62 Trametinib Dose Response (Figure S1)

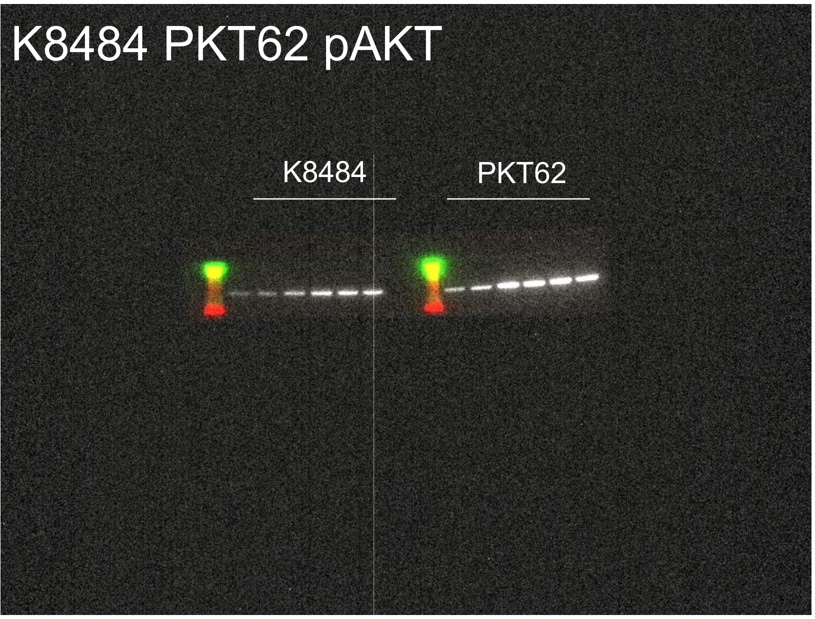

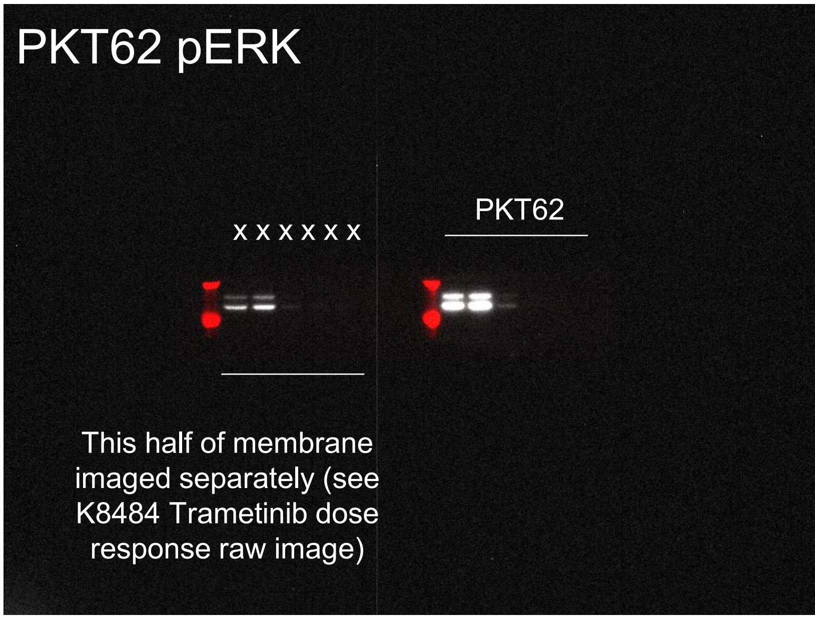

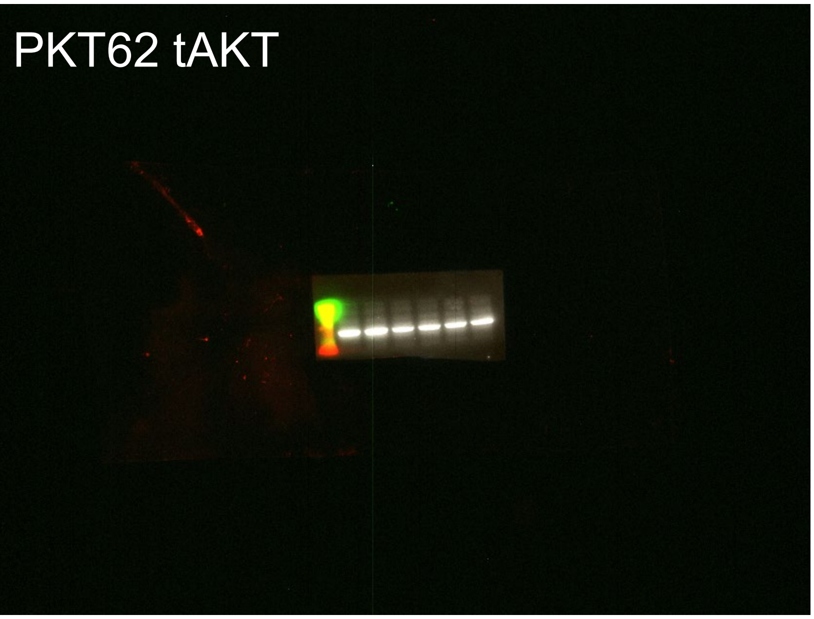

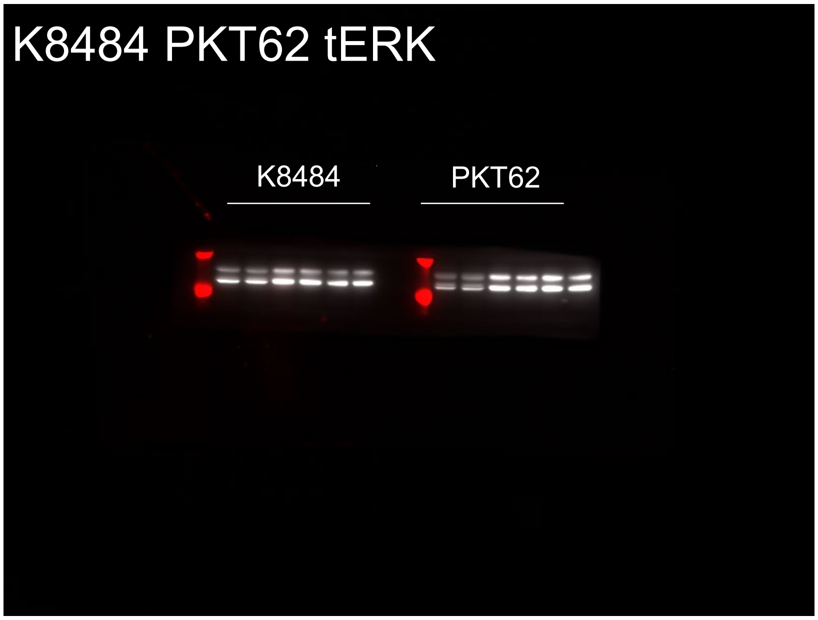

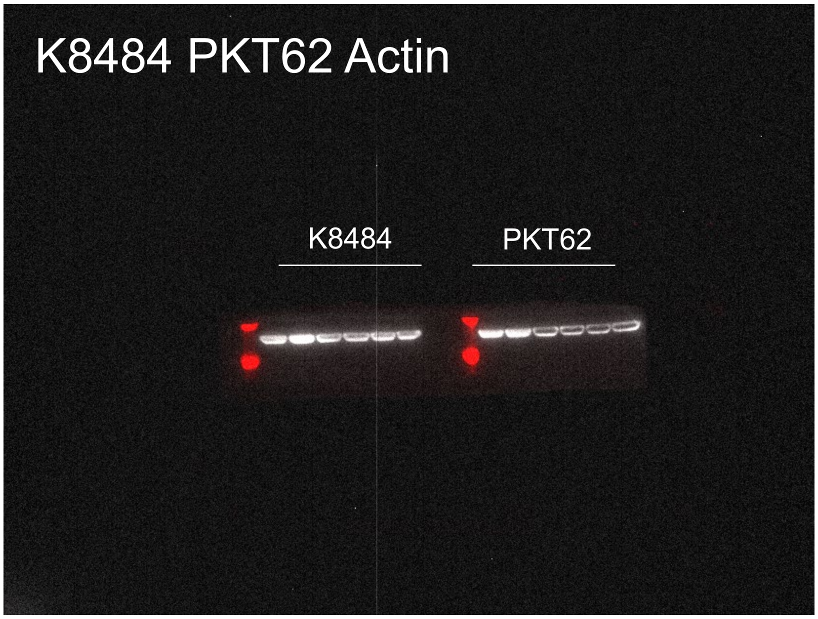

MiaPaCa-2 Trametinib Dose Response (Figure S1)

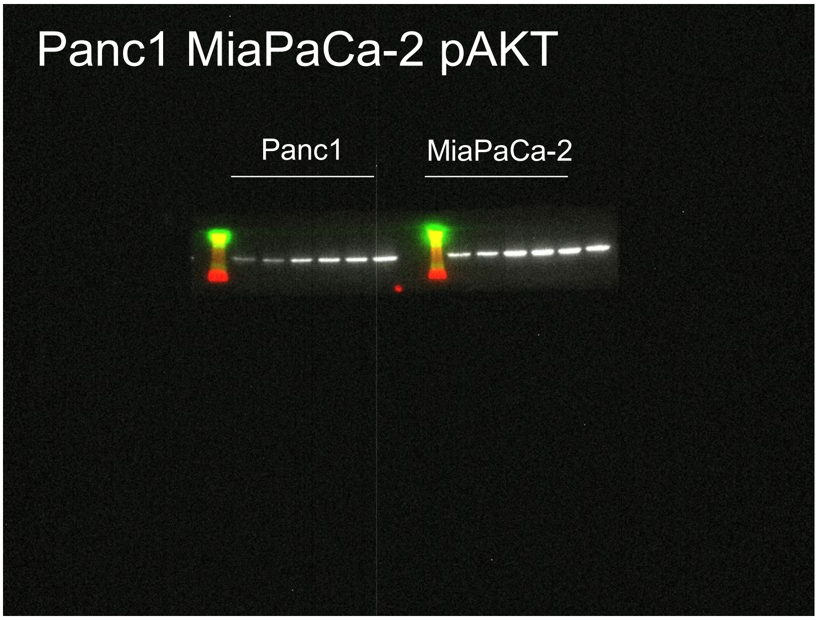

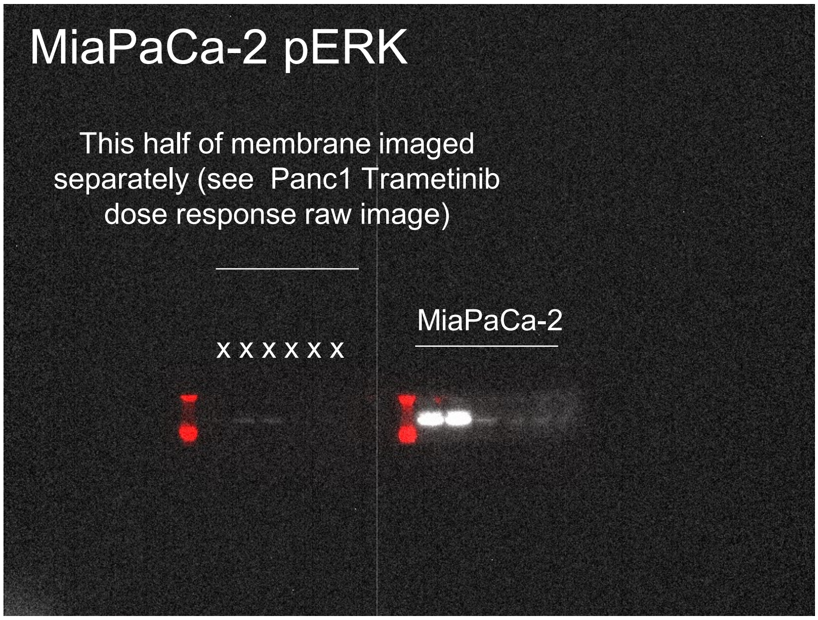

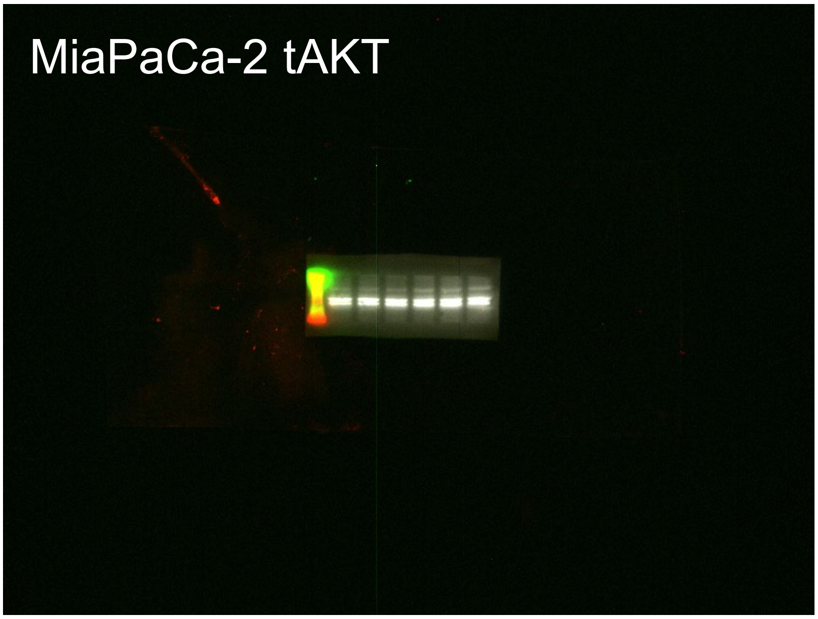

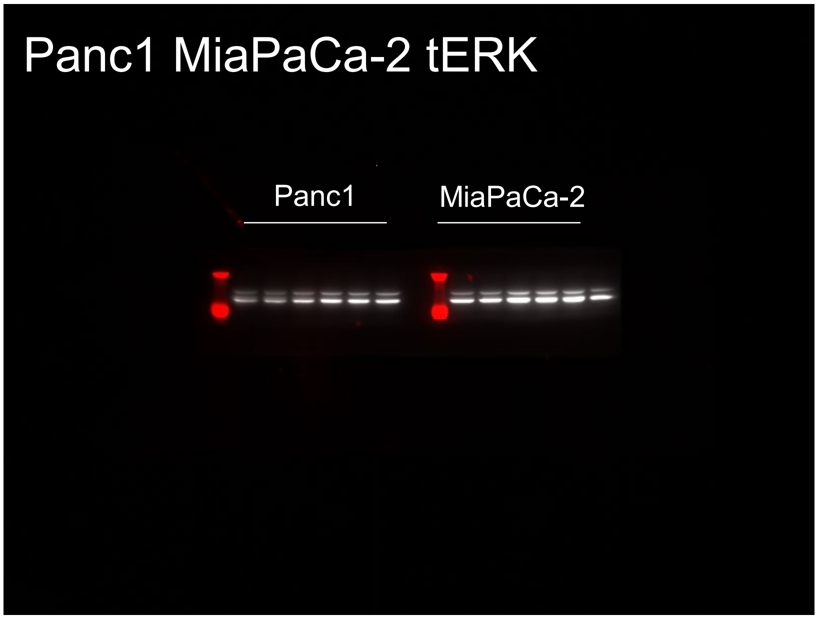

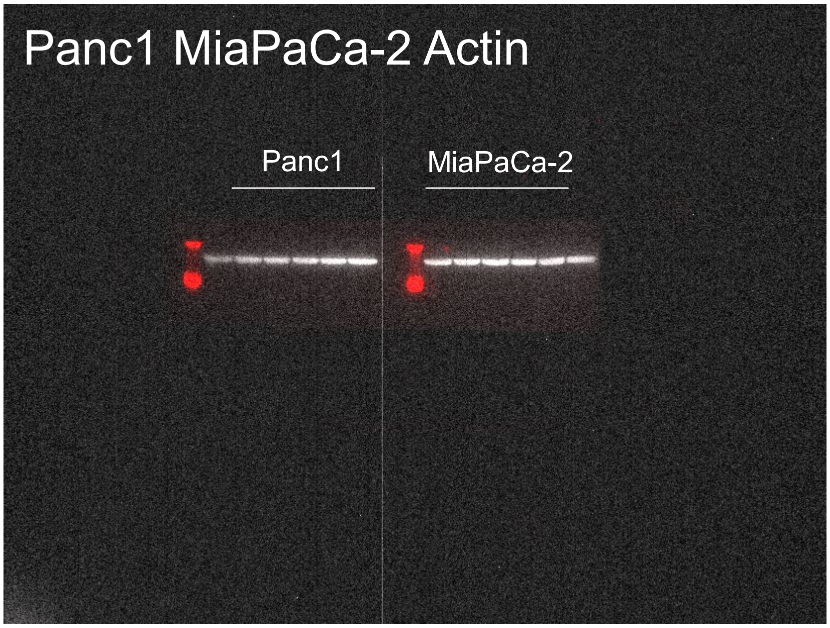

Panc1 Trametinib Dose Response (Figure S1)

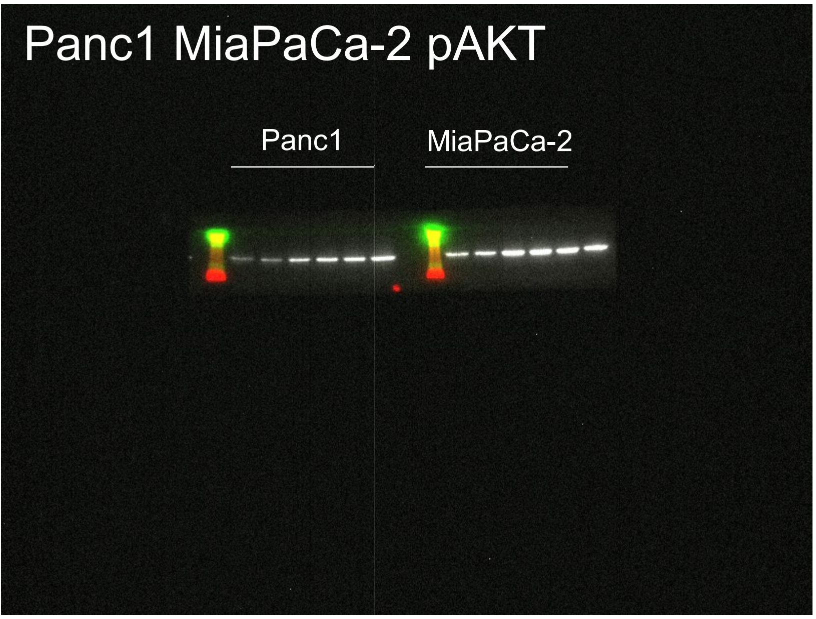

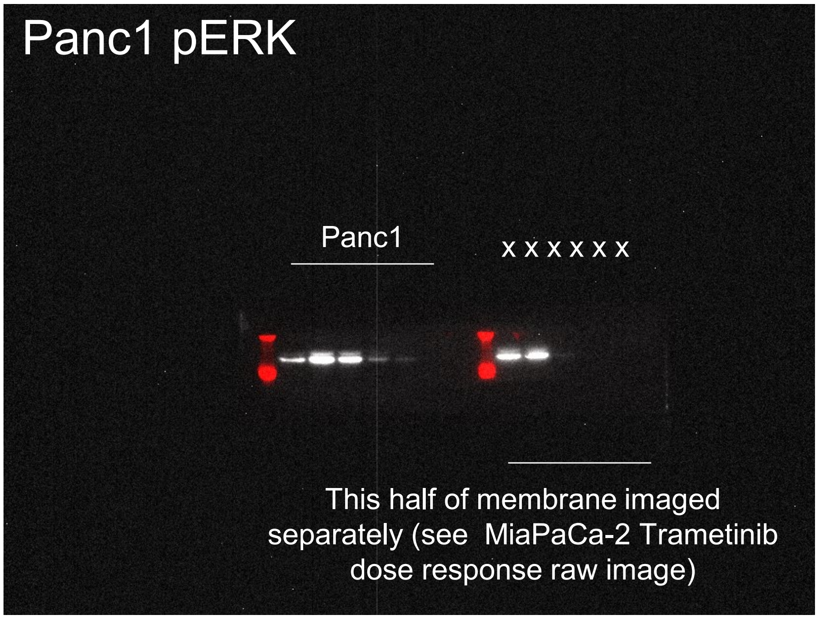

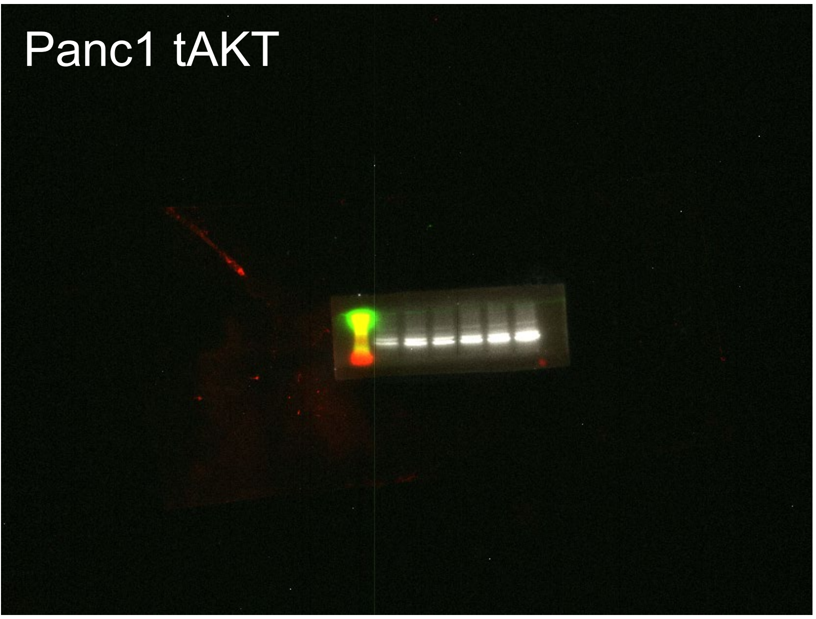

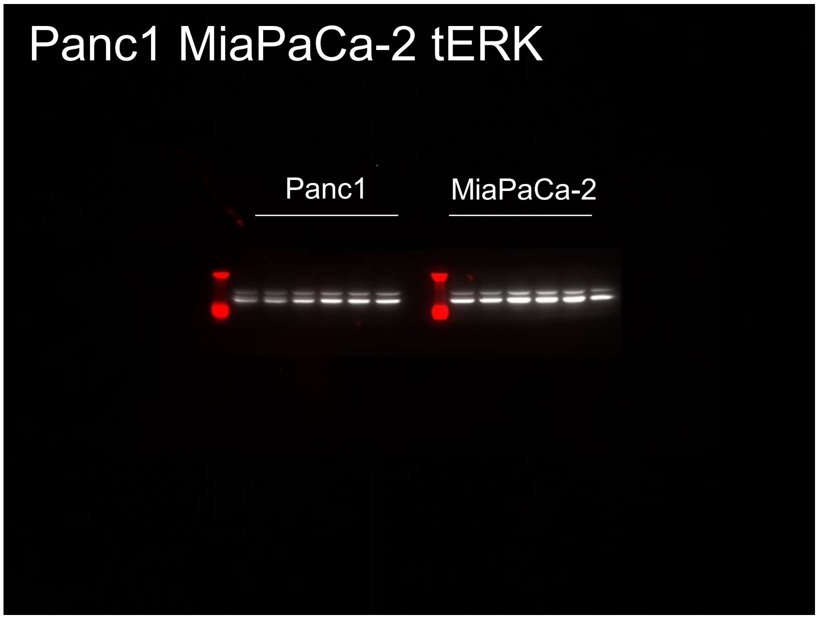

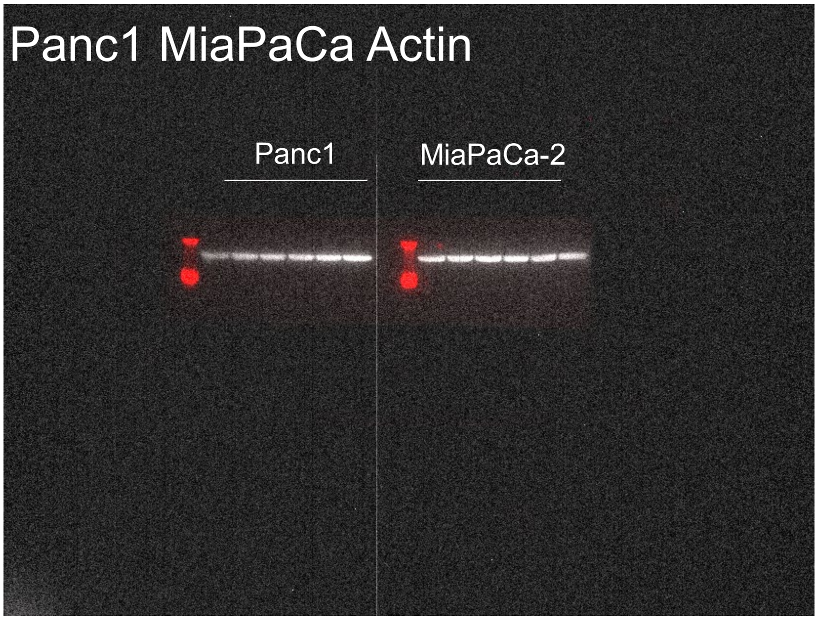

K8484 SHP099 Dose Response (Figure S1)

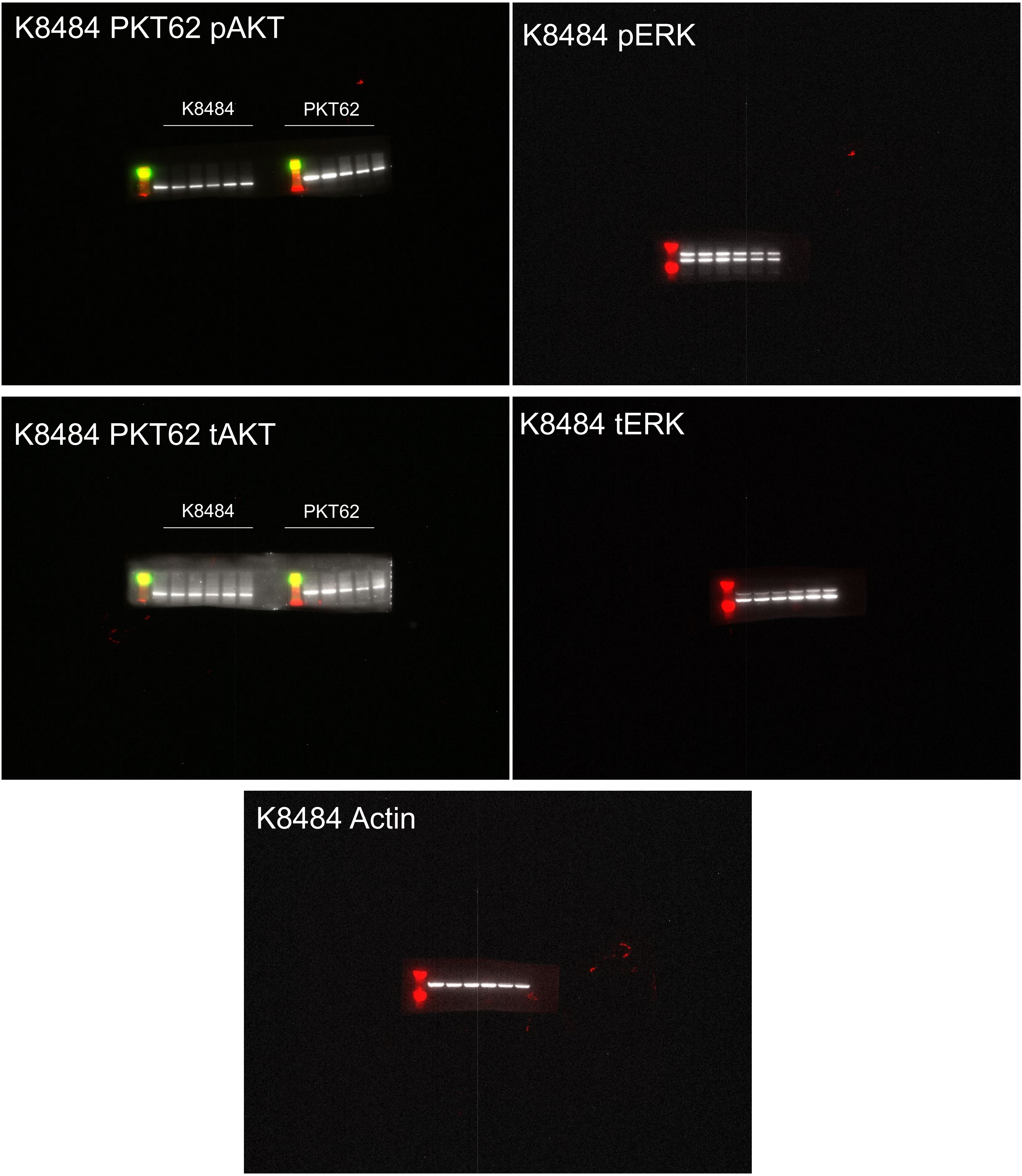

PKT62 SHP099 Dose Response (Figure S1)

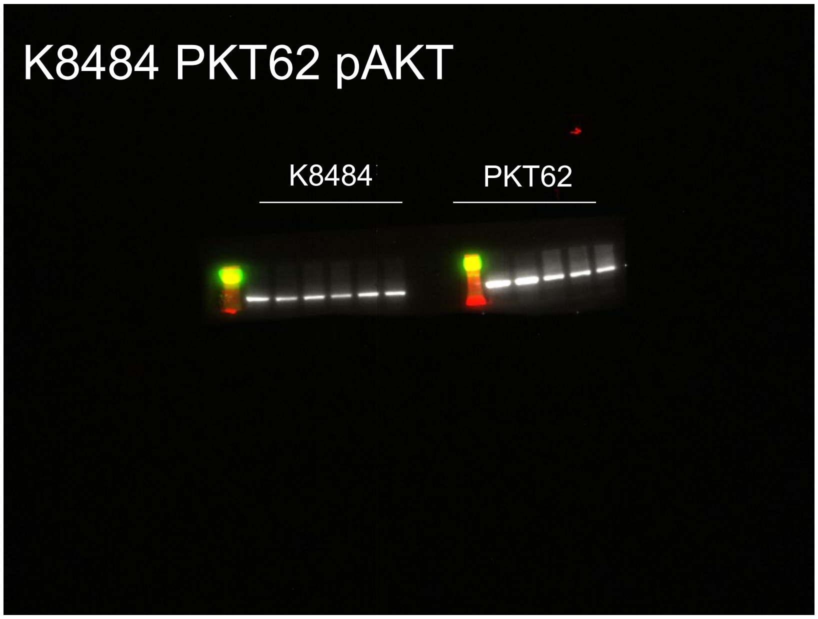

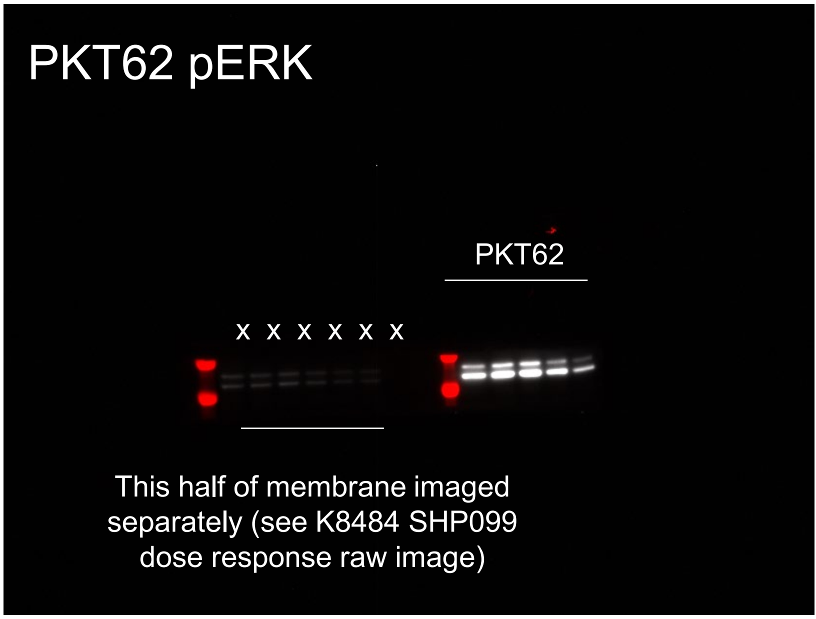

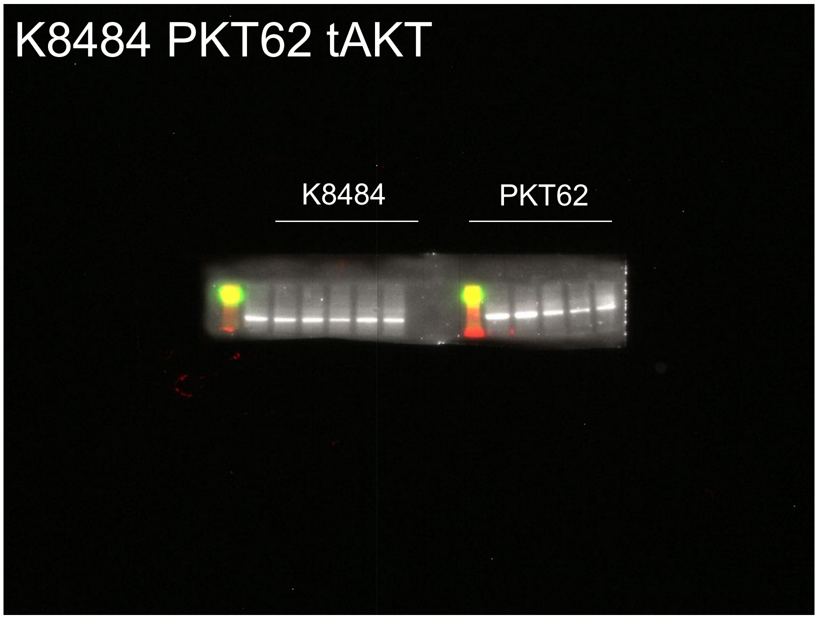

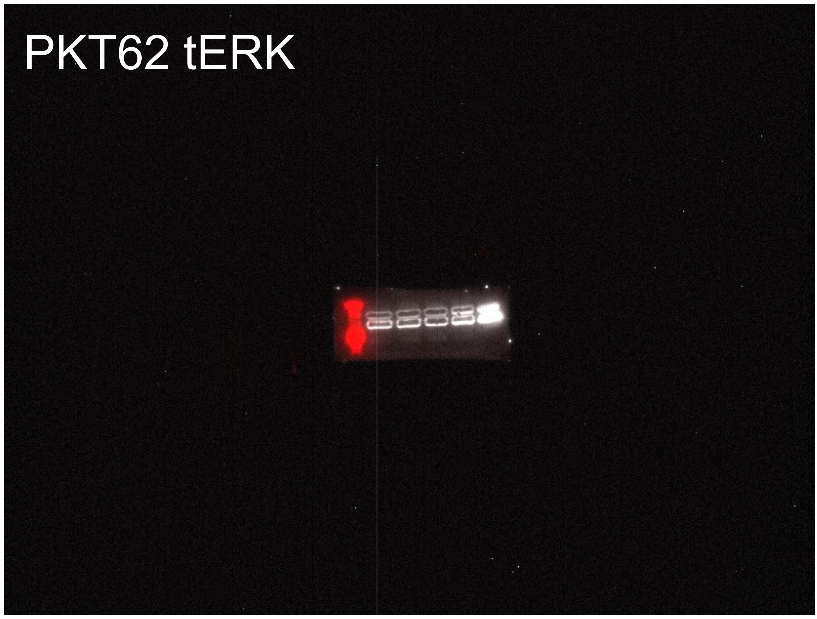

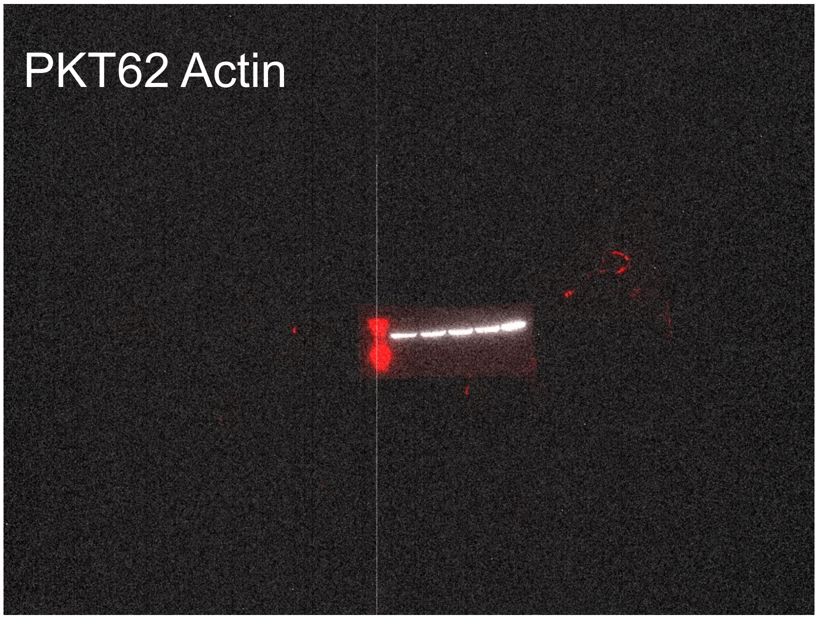

MiaPaCa-2 SHP099 Dose Response (Figure S1)

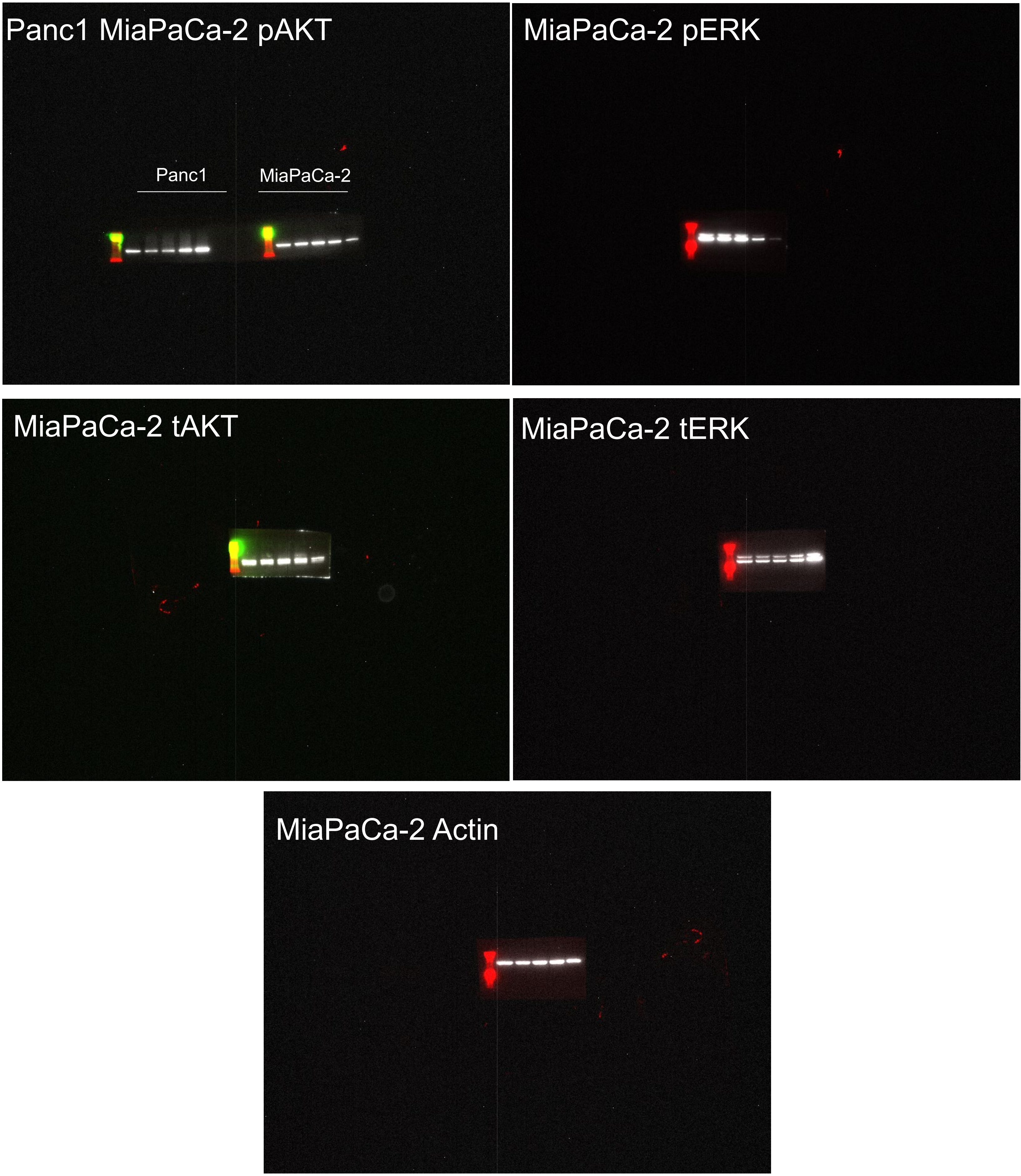

Panc1 SHP099 Dose Response (Figure S1)

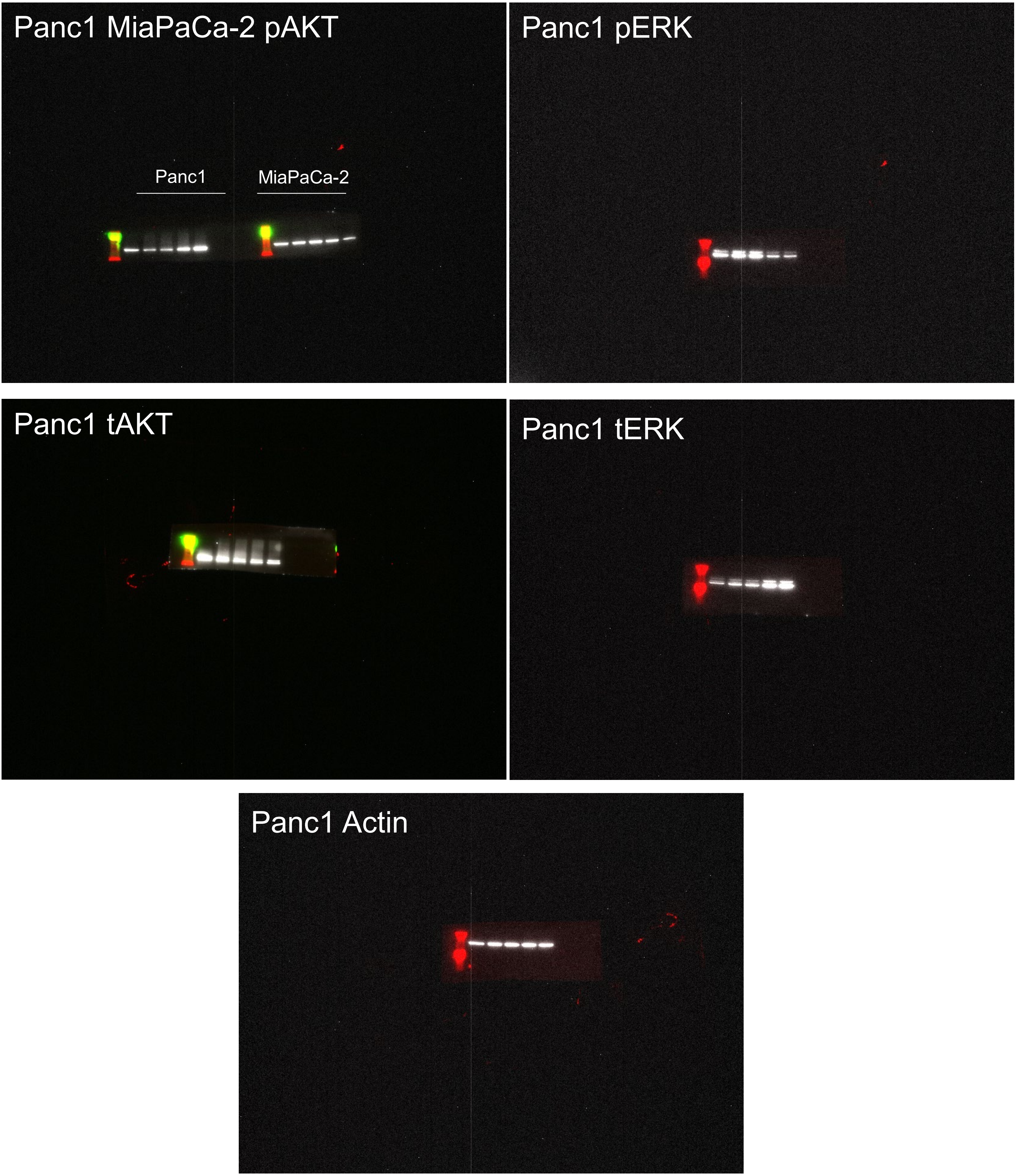

Tumor Size: Interaction effects model

The GLIMMIX Procedure

Group Indicators		
**Sl. No**	**Groups**	**Group_Ind**
**1**	Vehicle	1
**2**	Omipalisib	2
**3**	Trametinib	3
**4**	OmiTram	4
**5**	SHP099	5
**6**	OmiSHP	6

The following table shows the rate of tumor volume growth over time is statistically significantly different among the groups.

Type III Tests of Fixed Effects				
**Effect**	**Num DF**	**Den DF**	**F Value**	**Pr > F**
**Group_Ind**	5	26.53	1.41	0.2523
**Time**	9	231	20.43	<.0001
**Group_Ind*Time**	45	229.6	1.95	0.0008

The following table shows the rate of tumor growth differences between pairwise groups at several pairwise times.

Estimates					
**Label**	**Estimate**	**Standard Error**	**DF**	**t Value**	**Pr > |t|**
**Diff from Time 0 to Time 18 between Group 2 and Group 1**	-152.43	133.02	256.3	-1.15	0.2529
**Diff from Time 0 to Time 18 between Group 3 and Group 1**	-482.05	133.02	256.3	-3.62	0.0003
**Diff from Time 0 to Time 18 between Group 4 and Group 1**	-781.59	138.83	256.3	-5.63	<.0001
**Diff from Time 0 to Time 18 between Group 5 and Group 1**	-146.92	138.83	256.3	-1.06	0.2909
**Diff from Time 0 to Time 18 between Group 6 and Group 1**	-521.42	138.83	256.3	-3.76	0.0002
**Diff from Time 0 to Time 10 between Group 6 and Group 1**	-430.74	106.75	255.7	-4.03	<.0001
**Diff from Time 0 to Time 10 between Group 4 and Group 1**	-436.82	106.75	255.7	-4.09	<.0001
**Diff from Time 0 to Time 10 between Group 6 and Group 4**	6.0858	106.75	255.7	0.06	0.9546
**Diff from Time 0 to Time 18 between Group 6 and Group 4**	260.17	137.74	255.9	1.89	0.0600
**Diff from Time 0 to Time 18 between Group 1 and Group 4**	-781.59	138.83	256.3	-5.63	<.0001
**Diff from Time 0 to Time 18 between Group 2 and Group 4**	-629.16	131.87	255.9	-4.77	<.0001
**Diff from Time 0 to Time 18 between Group 3 and Group 4**	-299.54	131.87	255.9	-2.27	0.0239
**Diff from Time 0 to Time 18 between Group 5 and Group 4**	-634.67	137.74	255.9	-4.61	<.0001
**Diff from Time 0 to Time 18 between Group 6 and Group 4**	-260.17	137.74	255.9	-1.89	0.0600

Tumor Size: Interaction effects model

The GLIMMIX Procedure

The following table shows the differences in tumor volume between pairwise groups at several times.

Simple Effect Comparisons of Group_Ind*Time Least Squares Means By Time<br>Adjustment for Multiple Comparisons: Tukey-Kramer								
**Simple Effect Level**	**Group_Ind**	**_Group_Ind**	**Estimate**	**Standard Error**	**DF**	**t Value**	**Pr > |t|**	**Adj P**
**Time 2**	2	3	20.7776	160.00	33.77	0.13	0.8974	1.0000
**Time 2**	2	4	29.1192	167.81	33.77	0.17	0.8633	1.0000
**Time 2**	2	5	6.9015	167.81	33.77	0.04	0.9674	1.0000
**Time 2**	2	6	-45.2692	167.81	33.77	-0.27	0.7890	0.9998
**Time 2**	2	1	-52.9979	167.81	33.77	-0.32	0.7541	0.9996
**Time 2**	3	4	8.3416	167.81	33.77	0.05	0.9606	1.0000
**Time 2**	3	5	-13.8761	167.81	33.77	-0.08	0.9346	1.0000
**Time 2**	3	6	-66.0468	167.81	33.77	-0.39	0.6964	0.9988
**Time 2**	3	1	-73.7755	167.81	33.77	-0.44	0.6630	0.9979
**Time 2**	4	5	-22.2178	175.27	33.77	-0.13	0.8999	1.0000
**Time 2**	4	6	-74.3884	175.27	33.77	-0.42	0.6740	0.9982
**Time 2**	4	1	-82.1172	175.27	33.77	-0.47	0.6424	0.9972
**Time 2**	5	6	-52.1707	175.27	33.77	-0.30	0.7678	0.9997
**Time 2**	5	1	-59.8994	175.27	33.77	-0.34	0.7347	0.9994
**Time 2**	6	1	-7.7287	175.27	33.77	-0.04	0.9651	1.0000
**Time 4**	2	3	16.5715	160.00	33.77	0.10	0.9181	1.0000
**Time 4**	2	4	53.6219	167.81	33.77	0.32	0.7513	0.9996
**Time 4**	2	5	-21.0547	167.81	33.77	-0.13	0.9009	1.0000
**Time 4**	2	6	-3.3547	167.81	33.77	-0.02	0.9842	1.0000
**Time 4**	2	1	-68.1063	167.81	33.77	-0.41	0.6874	0.9986
**Time 4**	3	4	37.0504	167.81	33.77	0.22	0.8266	0.9999
**Time 4**	3	5	-37.6262	167.81	33.77	-0.22	0.8239	0.9999
**Time 4**	3	6	-19.9262	167.81	33.77	-0.12	0.9062	1.0000
**Time 4**	3	1	-84.6778	167.81	33.77	-0.50	0.6171	0.9960
**Time 4**	4	5	-74.6766	175.27	33.77	-0.43	0.6728	0.9982
**Time 4**	4	6	-56.9766	175.27	33.77	-0.33	0.7471	0.9995
**Time 4**	4	1	-121.73	175.27	33.77	-0.69	0.4921	0.9824
**Time 4**	5	6	17.7000	175.27	33.77	0.10	0.9202	1.0000
**Time 4**	5	1	-47.0516	175.27	33.77	-0.27	0.7900	0.9998

Tumor Size: Interaction effects model

The GLIMMIX Procedure

Simple Effect Comparisons of Group_Ind*Time Least Squares Means By Time<br>Adjustment for Multiple Comparisons: Tukey-Kramer								
Simple Effect Level	Group_Ind	_Group_Ind	Estimate	Standard Error	DF	t Value	Pr > |t|	Adj P
Time 4	6	1	-64.7516	175.27	33.77	-0.37	0.7141	0.9991
Time 6	2	3	64.3237	160.00	33.77	0.40	0.6902	0.9986
Time 6	2	4	114.46	167.81	33.77	0.68	0.4998	0.9838
Time 6	2	5	29.1784	167.81	33.77	0.17	0.8630	1.0000
Time 6	2	6	86.5163	167.81	33.77	0.52	0.6095	0.9955
Time 6	2	1	-131.56	167.81	33.77	-0.78	0.4385	0.9700
Time 6	3	4	50.1333	167.81	33.77	0.30	0.7670	0.9997
Time 6	3	5	-35.1454	167.81	33.77	-0.21	0.8354	0.9999
Time 6	3	6	22.1926	167.81	33.77	0.13	0.8956	1.0000
Time 6	3	1	-195.89	167.81	33.77	-1.17	0.2513	0.8519
Time 6	4	5	-85.2787	175.27	33.77	-0.49	0.6297	0.9966
Time 6	4	6	-27.9407	175.27	33.77	-0.16	0.8743	1.0000
Time 6	4	1	-246.02	175.27	33.77	-1.40	0.1695	0.7248
Time 6	5	6	57.3380	175.27	33.77	0.33	0.7456	0.9995
Time 6	5	1	-160.74	175.27	33.77	-0.92	0.3656	0.9418
Time 6	6	1	-218.08	175.27	33.77	-1.24	0.2220	0.8145
Time 8	2	3	71.7381	160.00	33.77	0.45	0.6568	0.9977
Time 8	2	4	163.46	167.81	33.77	0.97	0.3369	0.9257
Time 8	2	5	46.9131	167.81	33.77	0.28	0.7815	0.9998
Time 8	2	6	120.34	167.81	33.77	0.72	0.4782	0.9797
Time 8	2	1	-209.16	167.81	33.77	-1.25	0.2212	0.8134
Time 8	3	4	91.7217	167.81	33.77	0.55	0.5883	0.9941
Time 8	3	5	-24.8250	167.81	33.77	-0.15	0.8833	1.0000
Time 8	3	6	48.6012	167.81	33.77	0.29	0.7739	0.9997
Time 8	3	1	-280.90	167.81	33.77	-1.67	0.1034	0.5505
Time 8	4	5	-116.55	175.27	33.77	-0.66	0.5106	0.9855
Time 8	4	6	-43.1206	175.27	33.77	-0.25	0.8072	0.9999
Time 8	4	1	-372.62	175.27	33.77	-2.13	0.0409	0.2776
Time 8	5	6	73.4262	175.27	33.77	0.42	0.6779	0.9983
Time 8	5	1	-256.07	175.27	33.77	-1.46	0.1533	0.6894
Time 8	6	1	-329.50	175.27	33.77	-1.88	0.0688	0.4172

Tumor Size: Interaction effects model

The GLIMMIX Procedure

Simple Effect Comparisons of Group_Ind*Time Least Squares Means By Time<br>Adjustment for Multiple Comparisons: Tukey-Kramer								
**Simple Effect Level**	**Group_Ind**	**_Group_Ind**	**Estimate**	**Standard Error**	**DF**	**t Value**	**Pr > |t|**	**Adj P**
**Time 10**	2	3	83.6460	160.00	33.77	0.52	0.6045	0.9952
**Time 10**	2	4	212.39	167.81	33.77	1.27	0.2143	0.8034
**Time 10**	2	5	56.0436	167.81	33.77	0.33	0.7405	0.9994
**Time 10**	2	6	170.50	167.81	33.77	1.02	0.3168	0.9123
**Time 10**	2	1	-237.14	167.81	33.77	-1.41	0.1668	0.7190
**Time 10**	3	4	128.74	167.81	33.77	0.77	0.4483	0.9727
**Time 10**	3	5	-27.6024	167.81	33.77	-0.16	0.8703	1.0000
**Time 10**	3	6	86.8498	167.81	33.77	0.52	0.6081	0.9955
**Time 10**	3	1	-320.79	167.81	33.77	-1.91	0.0644	0.3977
**Time 10**	4	5	-156.34	175.27	33.77	-0.89	0.3787	0.9481
**Time 10**	4	6	-41.8898	175.27	33.77	-0.24	0.8125	0.9999
**Time 10**	4	1	-449.53	175.27	33.77	-2.56	0.0149	0.1103
**Time 10**	5	6	114.45	175.27	33.77	0.65	0.5182	0.9867
**Time 10**	5	1	-293.19	175.27	33.77	-1.67	0.1036	0.5512
**Time 10**	6	1	-407.64	175.27	33.77	-2.33	0.0262	0.1880
**Time 12**	2	3	162.28	160.00	33.77	1.01	0.3177	0.9129
**Time 12**	2	4	278.41	167.81	33.77	1.66	0.1064	0.5603
**Time 12**	2	5	58.1859	167.81	33.77	0.35	0.7309	0.9993
**Time 12**	2	6	190.81	167.81	33.77	1.14	0.2635	0.8654
**Time 12**	2	1	-246.36	167.81	33.77	-1.47	0.1513	0.6849
**Time 12**	3	4	116.12	167.81	33.77	0.69	0.4937	0.9827
**Time 12**	3	5	-104.10	167.81	33.77	-0.62	0.5392	0.9895
**Time 12**	3	6	28.5294	167.81	33.77	0.17	0.8660	1.0000
**Time 12**	3	1	-408.64	167.81	33.77	-2.44	0.0203	0.1486
**Time 12**	4	5	-220.22	175.27	33.77	-1.26	0.2176	0.8082
**Time 12**	4	6	-87.5956	175.27	33.77	-0.50	0.6205	0.9961
**Time 12**	4	1	-524.77	175.27	33.77	-2.99	0.0051	0.0357
**Time 12**	5	6	132.62	175.27	33.77	0.76	0.4545	0.9743
**Time 12**	5	1	-304.55	175.27	33.77	-1.74	0.0914	0.5084
**Time 12**	6	1	-437.17	175.27	33.77	-2.49	0.0177	0.1301
**Time 14**	2	3	197.71	160.00	33.77	1.24	0.2251	0.8189

Tumor Size: Interaction effects model

The GLIMMIX Procedure

Simple Effect Comparisons of Group_Ind*Time Least Squares Means By Time Adjustment for Multiple Comparisons: Tukey-Kramer								
Simple Effect Level	Group_Ind	_Group_Ind	Estimate	Standard Error	DF	t Value	Pr > |t|	Adj P
Time 14	2	4	337.73	167.81	33.77	2.01	0.0522	0.3384
Time 14	2	5	52.0571	167.81	33.77	0.31	0.7583	0.9996
Time 14	2	6	162.58	167.81	33.77	0.97	0.3395	0.9273
Time 14	2	1	-290.88	167.81	33.77	-1.73	0.0921	0.5112
Time 14	3	4	140.02	167.81	33.77	0.83	0.4099	0.9608
Time 14	3	5	-145.65	167.81	33.77	-0.87	0.3915	0.9537
Time 14	3	6	-35.1330	167.81	33.77	-0.21	0.8354	0.9999
Time 14	3	1	-488.59	167.81	33.77	-2.91	0.0063	0.0451
Time 14	4	5	-285.67	175.27	33.77	-1.63	0.1124	0.5796
Time 14	4	6	-175.15	175.27	33.77	-1.00	0.3247	0.9178
Time 14	4	1	-628.61	175.27	33.77	-3.59	0.0010	0.0054
Time 14	5	6	110.52	175.27	33.77	0.63	0.5326	0.9886
Time 14	5	1	-342.94	175.27	33.77	-1.96	0.0587	0.3707
Time 14	6	1	-453.46	175.27	33.77	-2.59	0.0142	0.1046
Time 16	2	3	224.88	160.00	33.77	1.41	0.1690	0.7237
Time 16	2	4	501.79	167.81	33.77	2.99	0.0052	0.0361
Time 16	2	5	-19.0793	167.81	33.77	-0.11	0.9102	1.0000
Time 16	2	6	268.01	167.81	33.77	1.60	0.1196	0.6013
Time 16	2	1	-266.26	167.81	33.77	-1.59	0.1219	0.6082
Time 16	3	4	276.91	167.81	33.77	1.65	0.1082	0.5662
Time 16	3	5	-243.96	167.81	33.77	-1.45	0.1552	0.6939
Time 16	3	6	43.1328	167.81	33.77	0.26	0.7987	0.9998
Time 16	3	1	-491.14	167.81	33.77	-2.93	0.0061	0.0432
Time 16	4	5	-520.87	175.27	33.77	-2.97	0.0054	0.0380
Time 16	4	6	-233.78	175.27	33.77	-1.33	0.1912	0.7659
Time 16	4	1	-768.06	175.27	33.77	-4.38	0.0001	0.0003
Time 16	5	6	287.09	175.27	33.77	1.64	0.1107	0.5743
Time 16	5	1	-247.18	175.27	33.77	-1.41	0.1676	0.7208
Time 16	6	1	-534.28	175.27	33.77	-3.05	0.0045	0.0305
Time 18	2	3	324.74	160.00	33.77	2.03	0.0503	0.3288
Time 18	2	4	637.77	167.81	33.77	3.80	0.0006	0.0025

Tumor Size: Interaction effects model

The GLIMMIX Procedure

Simple Effect Comparisons of Group_Ind*Time Least Squares Means By Time Adjustment for Multiple Comparisons: Tukey-Kramer								
**Simple Effect Level**	**Group_Ind**	**_Group_Ind**	**Estimate**	**Standard Error**	**DF**	**t Value**	**Pr > |t|**	**Adj P**
**Time 18**	2	5	10.4781	167.81	33.77	0.06	0.9506	1.0000
**Time 18**	2	6	341.79	167.81	33.77	2.04	0.0496	0.3248
**Time 18**	2	1	-156.53	168.71	34.48	-0.93	0.3600	0.9390
**Time 18**	3	4	313.03	167.81	33.77	1.87	0.0708	0.4262
**Time 18**	3	5	-314.26	167.81	33.77	-1.87	0.0698	0.4216
**Time 18**	3	6	17.0523	167.81	33.77	0.10	0.9197	1.0000
**Time 18**	3	1	-481.27	168.71	34.48	-2.85	0.0073	0.0530
**Time 18**	4	5	-627.29	175.27	33.77	-3.58	0.0011	0.0056
**Time 18**	4	6	-295.98	175.27	33.77	-1.69	0.1005	0.5407
**Time 18**	4	1	-794.30	176.13	34.42	-4.51	<.0001	0.0001
**Time 18**	5	6	331.31	175.27	33.77	1.89	0.0673	0.4108
**Time 18**	5	1	-167.01	176.13	34.42	-0.95	0.3496	0.9334
**Time 18**	6	1	-498.32	176.13	34.42	-2.83	0.0077	0.0565
**Time 0**	2	3	-4.8800	160.00	33.77	-0.03	0.9758	1.0000
**Time 0**	2	4	8.6055	167.81	33.77	0.05	0.9594	1.0000
**Time 0**	2	5	15.9822	167.81	33.77	0.10	0.9247	1.0000
**Time 0**	2	6	-27.1986	167.81	33.77	-0.16	0.8722	1.0000
**Time 0**	2	1	-4.1025	167.81	33.77	-0.02	0.9806	1.0000
**Time 0**	3	4	13.4855	167.81	33.77	0.08	0.9364	1.0000
**Time 0**	3	5	20.8622	167.81	33.77	0.12	0.9018	1.0000
**Time 0**	3	6	-22.3185	167.81	33.77	-0.13	0.8950	1.0000
**Time 0**	3	1	0.7775	167.81	33.77	0.00	0.9963	1.0000
**Time 0**	4	5	7.3767	175.27	33.77	0.04	0.9667	1.0000
**Time 0**	4	6	-35.8040	175.27	33.77	-0.20	0.8394	1.0000
**Time 0**	4	1	-12.7080	175.27	33.77	-0.07	0.9426	1.0000
**Time 0**	5	6	-43.1807	175.27	33.77	-0.25	0.8069	0.9999
**Time 0**	5	1	-20.0846	175.27	33.77	-0.11	0.9094	1.0000
**Time 0**	6	1	23.0961	175.27	33.77	0.13	0.8959	1.0000
